# Supplementary material for: Strain level microbial detection and quantification with applications to single cell metagenomics
Source: Nat Commun. 2022 Oct 28;13:6430. doi: 10.1038/s41467-022-33869-7 (PMC9616933; doi:10.1038/s41467-022-33869-7)
Supplement: Supplementary file 1 — Supplementary Information [file 41467_2022_33869_MOESM1_ESM.pdf]

Supplementary Notes for Strain Level Microbial Detection and Quantification with Applications to Single Cell Metagenomics

Kaiyuan Zhu, Alejandro A. Schäffer, Welles Robinson, Junyan Xu, Eytan Ruppin, A. Funda Ergun, Yuzhen Ye, S. Cenk Sahinalp

1 Computing Unique and Doubly-Unique Substrings

1.1 Notation and definitions

Let $s = s_1\$_1 \circ \cdots \circ s_m\$_m$ denote the string obtained by concatenating the input reference genomes $s_i \in \mathcal{S}$ and let $M = |s| = \sum_i |s_i|$ denote its length. A substring of s is a string in the form $s[l : r] = s[l]s[l+1]\cdots s[r]$. With a slight abuse of notation we denote by $s_i[l : r]$ not the actual substring of s_i including its lth to r^{th} symbols, but rather the substring of s including its lth to r^{th} symbols, with the provision that all these symbols are within the representation of s_i in s. We denote by an ℓ-mer a string of length ℓ. The suffix of s that starts at position i is denoted $\texttt{suf}[i] = s[i, \cdots, M]$. In what follows, we use the *generalized enhanced suffix array* of s that is composed of three parts. (i) The *suffix array* SA of s, which is comprised of the positions $1, 2, \cdots, M$, sorted in increasing lexicographical order of the corresponding suffixes $\texttt{suf}[i]$, $i = 1, 2, \cdots, M$. That is, $\texttt{SA}[i] = j$ indicates that $\texttt{suf}[j]$ is the i-th smallest suffix in lexicographical order. In addition, we denote $\texttt{SA}^{-1}[j] = i$ if $\texttt{SA}[i] = j$. (ii) The *longest common prefix array*, LCP, contains in its i-th position the length of the longest common prefix of $\texttt{suf}[\texttt{SA}[i]]$ and $\texttt{suf}[\texttt{SA}[i-1]]$, for $2 \leq i \leq M$ (and $\texttt{LCP}[1] = 0$). (iii) Finally, the *generalized suffix array* GSA contains the genome ID of each suffix $\texttt{suf}[\texttt{SA}[i]]$. All of the above arrays can be constructed in linear time: the first data structure that can be constructed in linear time, with the ability to determine whether a given substring is unique to a "document" (i.e. a genome in our context) in a collection of documents, and compute the shortest unique substring of a document that ends in a particular position, in time proportional to the substring length is the augmented suffix tree of Matias et al. [1]. Once an augmented suffix tree is computed, it can be trivially reduced to the above described enhanced suffix array in $O(M)$ time. The enhanced suffix array can also be constructed without the use of suffix trees to achieve a constant factor improvement in memory [2, 3].

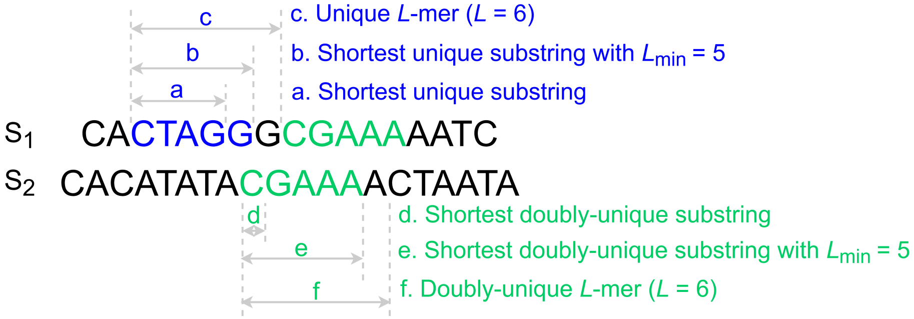

Supplementary Figure 1: Unique substrings, doubly-unique substrings, and their relationship with unique/doubly-unique L-mers.

We denote by $u_i(l, r)$ the substring $s_i[l : r]$ that is *unique* to genome s_i; formally, this indicates that there exists no substring $u_j(l', r')$ on any genome $s_j \neq s_i$ such that $u_i(l, r) = u_j(l', r')$. We call $u_i(l, r)$ a shortest unconstrained unique substring, if none of its substrings are unique. Similarly, we denote by $d_i(l, r)$ the substring $s_i[l : r]$ that is *doubly-unique* to genome s_i and one other genome, say s_j; formally, this indicates that there is exactly one genome s_j containing the substring $d_j(l', r')$, i.e., $s_i[l : r] = s_j[l' : r']$ for some l', r'.

Clearly, any superstring of a unique substring is still unique and any superstring of a doubly-unique substring is either unique or doubly-unique. We call $d_i(l, r)$ a shortest unconstrained doubly-unique substring of s_i and some other genome s_j, if none of its substrings are doubly-unique.

For our purposes (see the discussion in **Section CAMMiQ Index**, main text), we need to constrain the shortest unique and doubly-unique substrings with length upper bound $L_{\max}$ and lower bound $L_{\min}$. Under these constraints, we call any shortest unconstrained unique substring $u_i(l, r)$ a shortest unique substring if $L_{\max} \geq r - l + 1 > L_{\min}$. We also call a unique substring $u_i(l, r)$ a shortest unique substring if $r - l + 1 = L_{\min}$. Similarly, we call any shortest unconstrained doubly-unique substring $d_i(l, r)$ a shortest doubly-unique substring if $L_{\max} \geq r - l + 1 > L_{\min}$. Again, we call a doubly-unique substring $u_i(l, r)$ a shortest doubly-unique substring if $r - l + 1 = L_{\min}$ as well. We say an L-mer $s_i[l : l + L - 1]$ includes a unique substring $s_i[l' : r']$, or, conversely, a unique substring $s_i[l' : r']$ covers an L-mer $s_i[l : l + L - 1]$ if $l' \geq l$ and $r' \leq l + L - 1$. As such, we call an L-mer unique if it includes a unique substring. We can generalize these definitions to the notion of an L-mer including a doubly-unique substring, or conversely, a doubly-unique substring covering an L-mer, and thus making the L-mer itself doubly-unique - provided that it is not unique. See **Supplementary Figure 1** for a graphical illustration of the length bound on the shortest unique and doubly-unique substrings and their relationship to unique and doubly-unique L-mers.

1.2 Algorithmic framework to compute shortest unique substrings

It is quite simple to compute the shortest unique and doubly-unique substrings in $\mathcal{S}$ in $O(M)$ time by using the augmented suffix tree described in [1]. A similar running time can also be achieved through the use of a suffix array, as discussed by [4] for a single document (i.e. genome). We slightly generalize this to handle multiple genomes as follows. The key observation we use is that given a position l, the shortest unique or doubly-unique substring of s_i that starts at l (i.e. $u_i(l, r)$ or $d_i(l, r)$) is the shortest unique, or respectively doubly-unique prefix of $\texttt{suf}[l]$. In this way the problem can be reduced to searching for the longest common prefix of $\texttt{suf}[l]$ with any other suffix from another genome (i.e., any genome with ID $\neq \texttt{GSA}[\texttt{SA}^{-1}[l]]$) for each $1 \leq l \leq M$. Let $\text{lcp}(x, y)$ denote the longest common prefix of two suffices x and y; then we define:

$$\texttt{LCP}_u[i] = \max_{1 \leq j \leq M; \texttt{GSA}[j] \neq \texttt{GSA}[i]} \text{lcp}(\texttt{suf}[\texttt{SA}[i]], \texttt{suf}[\texttt{SA}[j]]) \quad (1)$$

and

$$\texttt{LCP}_d[i] = \max_{1 \leq j \leq M; \texttt{GSA}[j] \neq \texttt{GSA}[i], \texttt{GSA}[j']} \text{lcp}(\texttt{suf}[\texttt{SA}[i]], \texttt{suf}[\texttt{SA}[j]]) \quad (2)$$

where j' indicates a suffix $\texttt{suf}[j']$ with $\texttt{GSA}[j'] \neq \texttt{GSA}[i]$, which maximizes $\text{lcp}(\texttt{suf}[\texttt{SA}[i]], \texttt{suf}[\texttt{SA}[j']])$; note that any value of j' that maximizes $\text{lcp}(\texttt{suf}[\texttt{SA}[i]], \texttt{suf}[\texttt{SA}[j']])$ will imply the same value for $\texttt{LCP}_d[i]$. CAMMiQ maintains an array SU (of length M) such that $\texttt{SU}[r] = l$ if $u_i(l, r)$ is a shortest unique substring. To compute SU, each of its entries is initially set to 0 and for each $i = 1, \ldots M$, one entry of SU is updated as

$$\texttt{SU}[\texttt{SA}[i] + \texttt{LCP}_u[i]] \leftarrow \max\{\texttt{SU}[\texttt{SA}[i] + \texttt{LCP}_u[i]], \texttt{SA}[i]\} \quad (3)$$

Similarly, CAMMiQ maintains an array SD (again of length M) such that $\texttt{SD}[r] = l$ if $d_i(l, r)$ is a shortest doubly-unique substring. Again each entry of SD is initially set to 0 and then for each $i = 1, \ldots M$, one entry of SD is updated as

$$\texttt{SD}[\texttt{SA}[i] + \texttt{LCP}_d[i]] \leftarrow \max\{\texttt{SD}[\texttt{SA}[i] + \texttt{LCP}_d[i]], \texttt{SA}[i]\}. \quad (4)$$

The pseudocode to compute arrays SU and SD according to equations (3) and (4) respectively, given SA, $\texttt{LCP}_u$ and $\texttt{LCP}_d$ is given in Algorithm 1 below. As can be seen, the algorithm runs in $O(M)$ time.

Computing $\texttt{LCP}_u$ and $\texttt{LCP}_d$. Given $\texttt{GSA}[i_1, \cdots, i_2]$, a subarray of GSA, let $d_{\texttt{GSA}}(i_1, i_2)$ be the number of distinct genomes the entries in this subarray belong to, i.e. $d_{\texttt{GSA}}(i_1, i_2) = |\{\texttt{GSA}[i_1], \cdots, \texttt{GSA}[i_2]\}|$. We can now compute $\texttt{LCP}_u$ and $\texttt{LCP}_d$ in linear time as follows.

$$\texttt{LCP}_u[i] = \max \begin{cases} \min_{i^- < x \leq i} \texttt{LCP}[x], \text{ where } i^- = \max\{1 \leq i' < i\}, \text{ s.t. } d_{\texttt{GSA}}(i', i) \geq 2 \\ \min_{i < x \leq i^+} \texttt{LCP}[x], \text{ where } i^+ = \min\{i < i' \leq M\}, \text{ s.t. } d_{\texttt{GSA}}(i, i') \geq 2 \end{cases} \quad (5)$$

Algorithm 1 ShortestUniqueFromLCP($\texttt{SA}$, $\texttt{LCP}_u$, $\texttt{LCP}_d$, $L_{\max}$)

for i **from** 1 **to** M **do** //Initialize $\texttt{SU}$
  $\texttt{SU}[i] \leftarrow 0$
  $\texttt{SD}[i] \leftarrow 0$
end for
for i **from** 1 **to** M **do** //Update $\texttt{SU}$ according to (3) and $\texttt{SD}$ according to (4)
  if $\texttt{LCP}_u[i] < L_{\max}$ **then**
    $\texttt{SU}[\texttt{SA}[i] + \texttt{LCP}_u[i]] \leftarrow \max\{\texttt{SU}[\texttt{SA}[i] + \texttt{LCP}_u[i]], \texttt{SA}[i]\}$
  end if
  if $\texttt{LCP}_d[i] < L_{\max}$ **then**
    $\texttt{SD}[\texttt{SA}[i] + \texttt{LCP}_d[i]] \leftarrow \max\{\texttt{SD}[\texttt{SA}[i] + \texttt{LCP}_d[i]], \texttt{SA}[i]\}$
  end if
end for
return $\texttt{SU}, \texttt{SD}$

$$\texttt{LCP}_d[i] = \min \begin{cases} \max \begin{cases} \min\limits_{i^- < x \le i} \texttt{LCP}[x], \text{ where } i^- = \max\{1 \le i' < i\}, \text{ s.t. } d_{\texttt{GSA}}(i', i) \ge 2 \\ \min\limits_{i < x \le i^{2+}} \texttt{LCP}[x], \text{ where } i^{2+} = \min\{i < i' \le M\}, \text{ s.t. } d_{\texttt{GSA}}(i, i') \ge 3 \end{cases} \\ \max \begin{cases} \min\limits_{i^{2-} < x \le i} \texttt{LCP}[x], \text{ where } i^{2-} = \max\{1 \le i' < i\}, \text{ s.t. } d_{\texttt{GSA}}(i', i) \ge 3 \\ \min\limits_{i < x \le i^+} \texttt{LCP}[x], \text{ where } i^+ = \min\{i < i' \le M\}, \text{ s.t. } d_{\texttt{GSA}}(i, i') \ge 2 \end{cases} \end{cases} \quad (6)$$

Note that to introduce a minimum length constraint $L_{\min}$ on unique and doubly-unique substrings, each $\texttt{LCP}_u[i]$ is (re)set to $\max\{L_{\min} - 1, \texttt{LCP}_u[i]\}$ and respectively each $\texttt{LCP}_d[i]$ is (re)set to $\max\{L_{\min} - 1, \texttt{LCP}_d[i]\}$. Then, to ensure that each shortest doubly-unique substring occurs in exactly two genomes (and not one), we set $\texttt{LCP}_d[i] = \infty$ in case the above procedure ends up with $\texttt{LCP}_d[i] = \texttt{LCP}_u[i]$. See below for the proof of correctness and a running time analysis for the computation of $\texttt{LCP}_u$ and $\texttt{LCP}_d$.

1.3 Computing $\texttt{LCP}_u$ and $\texttt{LCP}_d$

In this section we show that $\texttt{SU}$ and $\texttt{SD}$ can be correctly constructed in $O(M)$ time. We start by showing that the definition of $\texttt{LCP}_u$ and $\texttt{LCP}_d$ in Equation (1) and Equation (2) can respectively lead to the shortest substrings occurring in at most one genome or two genomes. Then we give CAMMiQ's detailed implementation of Equation (5) and Equation (6) to compute the $\texttt{LCP}_u$ and $\texttt{LCP}_d$ arrays. Finally we give a running time analysis of this implementation.

First consider the content of $\texttt{SU}$ at the end of procedure ShortestUniqueFromLCP. To see the substring $s[l : r]$ corresponds to the r-th entry $\texttt{SU}[r] = l$ (where $l \ne 0$) in $\texttt{SU}$ is unique, meaning it only occurs in genome with ID $\texttt{GSA}[\texttt{SA}^{-1}[l]]$, assume that there is another genome $s_{i'}$ having the same substring $s[l', r'] = s[l : r]$ - this leads to a contradiction, since it implies that $\text{lcp}(\texttt{suf}[l], \texttt{suf}[l']) \ge r - l + 1$. However, due to the update rule of $\texttt{SU}$ and the definition of $\texttt{LCP}_u$, $\text{lcp}(\texttt{suf}[l], \texttt{suf}[l']) \le r - l$ for any $1 \le l' \le M$ satisfying $\texttt{suf}[l]$ and $\texttt{suf}[l']$ start on different genomes, namely $\texttt{GSA}[\texttt{SA}^{-1}[l]] \ne \texttt{GSA}[\texttt{SA}^{-1}[l']]$, which is a contradiction. Now, to see $s[l : r]$ is a shortest unique substring, i.e. no substring of $s[l : r]$ is unique to genome $\texttt{GSA}[\texttt{SA}^{-1}[l]]$, we show that any $s[l : r' < r]$ and $s[l' > l, r]$ occurs in one other genome $s_{i'}$. The former case is due to the definition of $\texttt{LCP}_u$ - there exists $\texttt{suf}[l']$ on genome $i' \ne \texttt{GSA}[\texttt{SA}^{-1}[l]]$ such that $\text{lcp}(\texttt{suf}[l], \texttt{suf}[l']) \ge r - l$, implying a substring $s[l' : l' + (r - l) - 1]$ identical to $s[l, r - 1]$; the later case is due to the update rule of $\texttt{SU}$ - if $s[l' > l, r]$ is also unique to genome $\texttt{GSA}[\texttt{SA}^{-1}[l]]$, then $\texttt{SU}[r]$ must be set to l' instead of l. Therefore, $s[l : r]$ is a shortest unique substring (to genome with ID $\texttt{GSA}[\texttt{SA}^{-1}[l]]$); on the other hand, if $s[l : r]$ is a shortest unique substring, then $\texttt{SU}[r]$ will maintain l after $\texttt{SU}$ is completely updated.

Now consider the content of $\texttt{SD}$ at the end of procedure ShortestUniqueFromLCP. We follow the above proof to show $s[l : r]$ is a shortest doubly-unique substring (to genome ID $\texttt{GSA}[\texttt{SA}^{-1}[l]]$ and possibly another genome i'). To see the substring $s[l : r]$ corresponds to the r-th entry $\texttt{SD}[r] = l$ (where $l \ne 0$) in $\texttt{SD}$ occurs in at most two genomes, with ID $\texttt{GSA}[\texttt{SA}^{-1}[l]]$ (and possibly i', any genome that $\texttt{suf}[l']$ belongs to, giving

the largest $\mathrm{lcp}(\texttt{suf}[l], \texttt{suf}[l']))$, we can assume there exists a thrid genome $s_{i''}$ having the same substring $s[l'', r''] = s[l, r] = s[l', r']$ and similarly obtain a contradiction. Note that according to (2), it is possible to have $\texttt{LCP}_d[\texttt{SA}^{-1}[l]] \geq \texttt{LCP}_u[\texttt{SA}^{-1}[l]]$ and in this case $s[l : r]$ is a unique substring that occurs only in genome $\texttt{GSA}[\texttt{SA}^{-1}[l]]$. If $\texttt{LCP}_d[\texttt{SA}^{-1}[l]] < \texttt{LCP}_u[\texttt{SA}^{-1}[l]]$ on the other hand, then $s[l : r]$ must occur in exactly two genomes, since SD is updated according to $\texttt{LCP}_d$ and we can find another suffix of s whose length-$(r - l + 1)$ $(r - l + 1 \leq \texttt{LCP}_d[\texttt{SA}^{-1}[l]])$ prefix is identical to $s[l : r]$. In addition, to see $s[l : r]$ is a shortest doubly-unique substring, meaning no substring of $s[l : r]$ occurs only in genome $\texttt{GSA}[\texttt{SA}^{-1}[l]]$ and i', we can similarly show that any $s[l : r' < r]$ and $s[l' > l, r]$ can be found in a third genome $s_{i''}$, regardless whether $\texttt{LCP}_d[\texttt{SA}^{-1}[l]] = \texttt{LCP}_u[\texttt{SA}^{-1}[l]]$ or $\texttt{LCP}_d[\texttt{SA}^{-1}[l]] < \texttt{LCP}_u[\texttt{SA}^{-1}[l]]$.

As a result of the above observations we can now formally state the following lemma.

Lemma 1. *(i)After updating SU according to (3), $SU[r] = l \neq 0$ implies that $s[l : r]$ is a shortest unique substring to genome $GSA[SA^{-1}[l]]$;*
(ii)After updating SD according to (4) $SD[r] = l \neq 0$ implies that $s[l : r]$ is a shortest doubly-unique substring to genome $GSA[SA^{-1}[l]]$ and $i' = GSA[SA^{-1}[l']]$ where $suf[l']$ gives the largest $lcp(suf[l], suf[l'])$).

Furthermore, it's also clear that all unique substrings $s[l : r]$ are stored in SU and all doubly-unique substrings $s[l : r]$ are stored in SD, as we have considered the suffix of s starting with every possible l.

Both $\texttt{LCP}_u$ and $\texttt{LCP}_d$ can be modified to incorporate the minimum length constraint $L_{\min}$ on unique/doubly-unique substrings. By setting $\texttt{LCP}_u[i]$ to $\max\{L_{\min} - 1, \texttt{LCP}_u[i]\}$ for each entry $1 \leq i \leq M$, the corresponding substrings maintained in SU should also be unique, and with minimum length $L_{\min}$. One should be careful however when dealing with $\texttt{LCP}_d$: if $\texttt{LCP}_u[i] \leq L_{\min} - 1$, then the corresponding substring $s[\texttt{SA}[i], \texttt{SA}[i] + \texttt{LCP}_u[i]]$ occurs in only one genome. Therefore we set $\texttt{LCP}_d[i]$ to ∞ (meaning it's not considered) if $\texttt{LCP}_u[i] \leq L_{\min} - 1$ or $\texttt{LCP}_d[i] \geq \texttt{LCP}_u[i]$; and to $\max\{L_{\min} - 1, \texttt{LCP}_d[i]\}$ otherwise (this can be done by first set each $\texttt{LCP}_u[i]$ to $\max\{L_{\min} - 1, \texttt{LCP}_u[i]\}$ and $\texttt{LCP}_d[i]$ to $\max\{L_{\min} - 1, \texttt{LCP}_d[i]\}$, and then set each $\texttt{LCP}_d[i]$ to ∞ if $\texttt{LCP}_d[i] \geq \texttt{LCP}_u[i]$), which ensures the corresponding substrings maintained in SD are doubly-unique, and with minimum length $L_{\min}$.

With the correctness of (3) and (4) in mind, our next concern is how to actually compute $\texttt{LCP}_u$ and $\texttt{LCP}_d$ based on their definitions. In the following we show that (5) and (6) correctly implement (1) and (2), without considering the borderline cases (i.e., for $i = 1$ or $i = M$; to handle these cases we can set $\texttt{GSA}[0] = \texttt{GSA}[M + 1] = 0$ and ignore i^{2-} when $i = 1$ and i^{2+} when $i = M$).

Lemma 2. *For any $1 \leq i \leq M$,*

$$(i)LCP_u[i] = \max \begin{cases} \min\limits_{i^- < x \leq i} LCP[x], \text{ where } i^- = \max\{1 \leq i' < i\}, \text{ s.t. } d_{GSA}(i', i) \geq 2 \\ \min\limits_{i < x \leq i^+} LCP[x], \text{ where } i^+ = \min\{i < i' \leq M\}, \text{ s.t. } d_{GSA}(i, i') \geq 2 \end{cases}$$

$$(ii)LCP_d[i] = \min \begin{cases} \max \begin{cases} \min\limits_{i^- < x \leq i} LCP[x], \text{ where } i^- = \max\{1 \leq i' < i\}, \text{ s.t. } d_{GSA}(i', i) \geq 2 \\ \min\limits_{i < x \leq i^{2+}} LCP[x], \text{ where } i^{2+} = \min\{i < i' \leq M\}, \text{ s.t. } d_{GSA}(i, i') \geq 3 \end{cases} \\ \max \begin{cases} \min\limits_{i^{2-} < x \leq i} LCP[x], \text{ where } i^{2-} = \max\{1 \leq i' < i\}, \text{ s.t. } d_{GSA}(i', i) \geq 3 \\ \min\limits_{i < x \leq i^+} LCP[x], \text{ where } i^+ = \min\{i < i' \leq M\}, \text{ s.t. } d_{GSA}(i, i') \geq 2 \end{cases} \end{cases}$$

where $d_{GSA}(i_1, i_2) = |\{GSA[i_1], \cdots, GSA[i_2]\}|$.

Proof. Let $\mathrm{lcp}(i, j)$ denote $\mathrm{lcp}(\texttt{suf}[i], \texttt{suf}[j])$ for short. We utilize the properties of SA and LCP array: (a) the longest common prefix of two suffices $\texttt{suf}[i]$ and $\texttt{suf}[j]$ (assume $\texttt{suf}[i]$ is lexicographically smaller than $\texttt{suf}[j]$) is $\mathrm{lcp}(i, j) = \min\{\texttt{LCP}[x] \mid x \in [\texttt{SA}^{-1}[i]+1, \texttt{SA}^{-1}[j]]\}$; also we have (b) $\mathrm{lcp}(i, j) \geq \mathrm{lcp}(\texttt{SA}[\texttt{SA}^{-1}[i]-1], j)$ and $\mathrm{lcp}(i, j) \geq \mathrm{lcp}(i, \texttt{SA}[\texttt{SA}^{-1}[j] + 1])$. (i) follows immediately from these properties:

$$\texttt{LCP}_u[i] = \max\{\mathrm{lcp}(\texttt{SA}[i], \texttt{SA}[i^+]), \mathrm{lcp}(\texttt{SA}[i^-], \texttt{SA}[i])\}.$$

To see (ii), we consider three cases:

- If $\text{lcp}(\mathtt{SA}[i], \mathtt{SA}[i^+]) = \text{lcp}(\mathtt{SA}[i^-], \mathtt{SA}[i])$, then $\mathtt{LCP}_d[i] = \text{lcp}(\mathtt{SA}[i], \mathtt{SA}[i^+]) = \text{lcp}(\mathtt{SA}[i^-], \mathtt{SA}[i])$, due to (b). (ii) holds in this case by applying (a).
- If $\text{lcp}(\mathtt{SA}[i], \mathtt{SA}[i^+]) < \text{lcp}(\mathtt{SA}[i^-], \mathtt{SA}[i])$, then $\mathtt{LCP}_d[i] = \max\{\text{lcp}(\mathtt{SA}[i], \mathtt{SA}[i^+]), \text{lcp}(\mathtt{SA}[i^{2-}], \mathtt{SA}[i])\}$ due to (b). Also $\mathtt{LCP}_d[i] \leq \text{lcp}(\mathtt{SA}[i^-], \mathtt{SA}[i]) = \max\{\text{lcp}(\mathtt{SA}[i], \mathtt{SA}[i^{2+}]), \text{lcp}(\mathtt{SA}[i^-], \mathtt{SA}[i])\}$ since $\text{lcp}(\mathtt{SA}[i], \mathtt{SA}[i^{2+}]) \leq \text{lcp}(\mathtt{SA}[i], \mathtt{SA}[i^+]) < \text{lcp}(\mathtt{SA}[i^-], \mathtt{SA}[i])$. (ii) therefore holds by applying (a).
- If $\text{lcp}(\mathtt{SA}[i], \mathtt{SA}[i^+]) > \text{lcp}(\mathtt{SA}[i^-], \mathtt{SA}[i])$, then we have similarly $\mathtt{LCP}_d[i] = \max\{\text{lcp}(\mathtt{SA}[i], \mathtt{SA}[i^{2+}]), \text{lcp}(\mathtt{SA}[i^-], \mathtt{SA}[i])\}$ due to (b) and $\mathtt{LCP}_d[i] \leq \text{lcp}(\mathtt{SA}[i], \mathtt{SA}[i^+]) = \max\{\text{lcp}(\mathtt{SA}[i], \mathtt{SA}[i^+]), \text{lcp}(\mathtt{SA}[i^{2-}], \mathtt{SA}[i])\}$ since $\text{lcp}(\mathtt{SA}[i^{2-}], \mathtt{SA}[i]) \leq \text{lcp}(\mathtt{SA}[i^-], \mathtt{SA}[i]) < \text{lcp}(\mathtt{SA}[i], \mathtt{SA}[i^+])$. Again we can see (ii) by applying (a), which completes the proof.

□

Next we give the pseudocode to update $\mathtt{LCP}_u$ and $\mathtt{LCP}_d$ based on (5) and (6) respectively in Algorithm 2 and 3, and show that they actually run in linear time.

Algorithm 2 $\text{LCP}_u\text{FromLCP}(\mathtt{GSA}, \mathtt{LCP}, L_{\min})$

$\mathtt{LCP}_u \leftarrow$ array of length M
$i \leftarrow 1$, minlcp $\leftarrow M$, next $\leftarrow 0$
while $i < M$ **do**
    while $i < M$ and $\mathtt{GSA}[i + \text{next}] = \mathtt{GSA}[i + \text{next} + 1]$ **do**
        next $\leftarrow$ next $+ 1$
    end while
    for j **from** next **to** 0 **do**
        minlcp $\leftarrow \min\{\text{minlcp}, \mathtt{LCP}[i + j + 1]\}$
        $\mathtt{LCP}_u[i + j] \leftarrow$ minlcp
    end for
    $i \leftarrow i + \text{next} + 1$
    minlcp $\leftarrow M$, next $\leftarrow 0$
end while
$i \leftarrow M$, minlcp $\leftarrow M$, next $\leftarrow 0$
while $i > 1$ **do**
    while $i > 1$ and $\mathtt{GSA}[i - \text{next}] = \mathtt{GSA}[i - \text{next} - 1]$ **do**
        next $\leftarrow$ next $+ 1$
    end while
    for j **from** next **to** 0 **do**
        minlcp $\leftarrow \min\{\text{minlcp}, \mathtt{LCP}[i - j]\}$
        $\mathtt{LCP}_u[i - j] \leftarrow \max\{\mathtt{LCP}_u[i - j], \text{minlcp}\}$
    end for
    $i \leftarrow i - \text{next} - 1$
    minlcp $\leftarrow M$, next $\leftarrow 0$
end while
for i **from** 1 **to** M **do**
    $\mathtt{LCP}_u[i] \leftarrow \max\{\mathtt{LCP}_u[i], L_{\min}\}$
end for
return $\mathtt{LCP}_u$

Algorithm 3 $\text{LCP}_d\text{FromLCP}(\mathtt{GSA}, \mathtt{LCP}, L_{\min})$

$\mathtt{LCP}_d \leftarrow$ array of length M
$\mathtt{LCP}_u \leftarrow \text{LCP}_u\text{FromLCP}(\mathtt{GSA}, \mathtt{LCP}, M, L_{\min})$
$\mathtt{LCP}_{d'} \leftarrow \text{LCP}_d\text{FromLCP+}(\mathtt{GSA}, \mathtt{LCP})$
$\mathtt{LCP}_{d''} \leftarrow \text{LCP}_d\text{FromLCP}-(\mathtt{GSA}, \mathtt{LCP})$
for i **from** 1 **to** M **do**
    $\mathtt{LCP}_d[i] \leftarrow \max\{\min\{\mathtt{LCP}_{d'}[i], \mathtt{LCP}_{d''}[i]\}, L_{\min}\}$
    if $\mathtt{LCP}_d[i] \geq \mathtt{LCP}_u[i]$ **then**
        $\mathtt{LCP}_d[i] \leftarrow \infty$
    end if
end for
return $\mathtt{LCP}_d$

Lemma 3. *Both LCP_uFromLCP and LCP_dFromLCP run in $O(M)$ time.*

Algorithm 4 LCP_dFromLCP+(GSA, LCP)

```
LCP*_d ← array of length M
i ← 1, minlcp ← M, next1 ← 0, next2 ← 1
while i < M do
    while i < M and GSA[i + next1] = GSA[i + next1 + 1] do
        next1 ← next1 + 1
    end while
    while i < M and GSA[i + next1 + next2] = GSA[i + next1 + next2 + 1] do
        next2 ← next2 + 1
    end while
    for j from next1 + next2 to 0 do
        minlcp ← min{minlcp, LCP[i + j + 1]}
        if j ≤ next1 then
            LCP*_d[i + j] ← minlcp
        end if
    end for
    i ← i + next1 + 1
    minlcp ← M, next1 ← 0, next2 ← 1
end while
i ← M, minlcp ← M, next1 ← 0
while i > 1 do
    while i > 1 and GSA[i − next1] = GSA[i − next1 − 1] do
        next1 ← next1 + 1
    end while
    for j from next1 to 0 do
        minlcp ← min{minlcp, LCP[i − j]}
        LCP*_d[i − j] ← max{LCP*_d[i − j], minlcp}
    end for
    i ← i − next1 − 1
    minlcp ← M, next1 ← 0
end while
return LCP*_d
```

Proof. Through a simple aggregate analysis, we can see that LCP_uFromLCP visits each entry of GSA, LCP and $\texttt{LCP}_u$ 2 times; LCP_dFromLCP+ (Algorithm 4) and LCP_dFromLCP− (Algorithm 5) both visit each entry of GSA, LCP 3 times and each entry of $\texttt{LCP}^*_d$ 2 times. □

Combining the above lemmata, we concluse that

Theorem 4. *Both SU and SD can be computed in in $O(M)$ time.*

2 Sparsifying unique substrings

Recall that $\mathcal{U}_i = \{u_{i,1}(l_1, r_1), u_{i,nu_i}(l_{nu_i}, r_{nu_i})\}$ defines either the collection of all shortest unique substrings or unique substrings with minimum length $L_{\min}$ on a given genome s_i (sorted by l_x, namely $l_1 \leq \cdots \leq l_{nu_i}$). In fact, the list of left indices $l_1, \cdots, l_{nu_i}$ are stored in the corresponding $r_1, \cdots, r_{nu_i}$ entries in SU array. Due to the minimum length constraint, no substring $u_{i,x} \in \mathcal{U}_i$ can be a substring of any other $u_{i,y} \in \mathcal{U}_i$ if they are not identical (in fact, there could be some $\{u_{i,x}(l_x, r_x) = \{u_{i,y}(l_y, r_y) \in \mathcal{U}_i$ for $x \neq y$). This makes $l_1 < \cdots < l_{nu_i}$ and $r_1 < \cdots < r_{nu_i}$. The goal of sampling unique substrings from $\mathcal{U}_i$ is to identify and maintain the smallest number of unique substrings such that they cover the same set of unique L-mers on s_i as $\mathcal{U}_i$ (if $L_{\max} = L$ then the sampled unique substrings should cover all unique L-mers).

Here we present the greedy sampling strategy implemented by CAMMiQ to sample unique substrings from $\mathcal{U}_i$. Denote by begin_i the beginning position of s_i in s and by $\mathcal{U}'_i$ the unique substrings already sampled

Algorithm 5 $\text{LCP}_d\text{FromLCP}-(\texttt{GSA}, \texttt{LCP})$

```
LCP*_d ← array of length M
i ← 1, minlcp ← M, next1 ← 0
while i < M do
    while i < M and GSA[i + next1] = GSA[i + next1 + 1] do
        next1 ← next1 + 1
    end while
    for j from next1 to 0 do
        minlcp ← min{minlcp, LCP[i + j + 1]}
        LCP*_d[i + j] ← minlcp
    end for
    i ← i + next1 + 1
    minlcp ← M, next1 ← 0
end while
i ← M, minlcp ← M, next1 ← 0, next2 ← 1
while i > 1 do
    while i > 1 and GSA[i − next1] = GSA[i − next1 − 1] do
        next1 ← next1 + 1
    end while
    while i > 1 and GSA[i − next1 − next2] = GSA[i − next1 − next2 − 1] do
        next2 ← next2 + 1
    end while
    for j from next1 + next2 to 0 do
        minlcp ← min{minlcp, LCP[i − j]}
        if j ≥ next2 then
            LCP*_d[i − j] ← max{LCP*_d[i − j], minlcp}
        end if
    end for
    i ← i − next1 − 1
    minlcp ← M, next1 ← 0, next2 ← 1
end while
return LCP*_d
```

(initially $\mathcal{U}'_i$ is empty). Starting with begin_i, consider every L-mer of s_i from left to right; if it does not include any unique substring, then ignore this L-mer; otherwise add its rightmost unique substring into $\mathcal{U}'_i$ and move to the next L-mer that does not include this substring until reaching the L-mer that ends at $\text{begin}_i + |s_i| - 1$. At the and of this, add the sampled unique substrings in $\mathcal{U}'_i$ to the hash table (and trie) described in **Section `CAMMiQ` Index**, main text.

In the following we show that the above greedy strategy obtains the smallest number of unique substrings that cover the same set of unique L-mers as $\mathcal{U}_i$, provided that each unique substring in $\mathcal{U}_i$ occurs only one time (i.e., any $u_{i,x} \in \mathcal{U}_i$ is not identical to another $u_{i,y} \in \mathcal{U}_i$ if $x \neq y$). As a result, the total number of unique substrings in $\mathcal{U}' = \cup_{i=1}^{m} \mathcal{U}'_i$ included in `CAMMiQ` index is also as small as possible.

Theorem 5. *If $u_{i,x} \in \mathcal{U}_i \neq u_{i,y} \in \mathcal{U}_i$ for $x \neq y$, then GreedySampling returns the smallest $\mathcal{U}'_i$ such that if an L-mer includes some $u_{i,x} \in \mathcal{U}_i$, then it also includes at least one $u_{i,x'} \in \mathcal{U}'_i$.*

Proof. Consider $\mathcal{U}'_i = \{u'_{i,1}(l'_1, r'_1), \cdots, u'_{i,|\mathcal{U}'_i|}(l'_{|\mathcal{U}'_i|}, r'_{|\mathcal{U}'_i|})\}$ that GreedySampling returns; also consider an alternative sample $\mathcal{U}''_i = \{u''_{i,1}(l''_1, r''_1), \cdots, u''_{i,|\mathcal{U}''_i|}(l''_{|\mathcal{U}''_i|}, r''_{|\mathcal{U}''_i|})\}$ of $\mathcal{U}_i$ that covers the same set of L-mers; assume both sets are sorted by the left indices ($l'_1 < \cdots < l'_{|\mathcal{U}'_i|}$; $l''_1 < \cdots < l''_{|\mathcal{U}''_i|}$). First, observe that $l'_1 \geq l''_1$ since $u'_{i,1}$ is the rightmost unique substring in $\mathcal{U}_i$ that is fully contained in the leftmost unique L-mer. As a consequence we must have $l'_2 \geq l''_2$ (and so on). Otherwise, if $l'_1 = l''_1$ then $u'_{i,2}(l'_2, r'_2)$ is not the rightmost unique substring in the next unique L-mer whose left index is greater than l'_1, and if $l'_1 > l''_1$ then there is some unique L-mer not covered by any unique substring in $\mathcal{U}''_i$. Therefore $|\mathcal{U}'_i| \leq |\mathcal{U}''_i|$ since there is a

Algorithm 6 GreedySampling($\mathcal{U}_i = \{u_{i,1}(l_1, r_1), u_{i,nu_i}(l_{nu_i}, r_{nu_i})\}$, L)

$\mathcal{U}_i' \leftarrow \emptyset$
cur $\leftarrow$ begin$_i$ // begin$_i$: the beginning position of s_i in s
$u_{i,\text{last}}(l_{\text{last}}, r_{\text{last}}) \leftarrow$ `NIL`
for x **from** 1 to nu_i **do**
  if $u_{i,\text{last}} \neq$ `NIL` and $r_x \geq \text{cur} + L$ **then**
    $\mathcal{U}_i' \leftarrow \mathcal{U}_i' \cup \{u_{i,\text{last}}(l_{\text{last}}, r_{\text{last}})\}$
    cur $\leftarrow l_{\text{last}} + 1$
  end if
  $u_{i,\text{last}}(l_{\text{last}}, r_{\text{last}}) \leftarrow u_{i,x}(l_x, r_x)$
end for
return $\mathcal{U}_i'$

injection between the elements in $\mathcal{U}_i'$ and $\mathcal{U}_i''$ until reaching the last unique L-mer on s_i. □

Corollary 6. *If $u_{i,x} \in \mathcal{U}_i \neq u_{i,y} \in \mathcal{U}_i$ for $x \neq y$ on each s_i, then $\mathcal{U}' = \cup_{i=1}^{m} \mathcal{U}_i'$ is the smallest set of unique substrings such that if an L-mer on s_i includes some $u_{i,x} \in \mathcal{U}_i$, then it also includes at least one $u_{i,x'} \in \mathcal{U}_i'$.*

We note that the greedy sampling strategy works in practice even if there are actually unique substrings occurring more than once in a given genome, meaning $u_{i,x} = u_{i,y} \in \mathcal{U}_i$ for some $y > x$, leading to $\mathcal{U}_i'$ (and thus $\mathcal{U}'$) being close to optimality, since these unique substrings would constitute a very small proportion ($\leq 0.1\%$) with the default minimum length $L_{\min} = 26$ of unique substrings. This gives significantly smaller indices than alternative k-mer based tools, and results in an integer program with a small number of constraints.

We applied the above strategy to sample doubly-unique substrings in $\mathcal{D}_i$, to obtain the minimum size $\mathcal{D}_i'$ for each genome s_i so that the aggregate set of doubly-unique substrings $\mathcal{D}'$ is at most twice the optimal, provided that each doubly-unique substring occurs once in each of the corresponding genomes.

Corollary 7. *If $d_{i,x} \in \mathcal{D}_i \neq d_{i,y} \in \mathcal{D}_i$ for $x \neq y$ on each s_i, then $\mathcal{D}' = \cup_{i=1}^{m} \mathcal{D}_i'$ is at most twice as large as the smallest set of doubly-unique substrings such that if an L-mer on s_i includes some $d_{i,x} \in \mathcal{D}_i$, then it also includes at least one $d_{i,x'} \in \mathcal{D}_i'$.*

3 Query Processing Stage 1: Preprocessing the Reads

Let $\mathcal{U}$ and $\mathcal{D}$ be the collection(s) of indexed unique and doubly-unique substrings, respectively. Remind that we maintain a counter $c(u_i)$ for each unique substring $u_i \in \mathcal{U}$, and a counter $c(d_i)$ for each unique substring $d_i \in \mathcal{D}$, indicating how many reads in a query $\mathcal{Q}$ include u_i and d_i. To process each read $r_j \in \mathcal{Q}$, we follow the following procedure.

1. Identify the set $U_j = \{u_{j,1}, \ldots u_{j,\ell}\} \subset \mathcal{U}$ of unique substrings in r_j; similarly identify the set $D_j = \{d_{j,1}, \ldots d_{j,\ell'}\} \subset \mathcal{D}$ of doubly-unique substrings in r_j.
2. (a) If $U_j = D_j = \emptyset$ then discard r_j.
   (b) If $U_j \neq \emptyset$ but it includes a pair of unique substrings $u_{j,i}$ and $u_{j,i'}$ that originate from different genomes, then discard r_j.
   (c) If $U_j \neq \emptyset$ and all its unique substrings originate from the same genome s_k, however D_j includes a substring $d_{j,i}$ that cannot originate from s_k, then again discard r_j.
   (d) If $U_j = \emptyset$ and the intersection of the set of genomes from where the substrings in D_j can originate is empty, then also discard r_j.
   (e) If on the other hand,
      i. $U_j \neq \emptyset$, all its unique substrings originate from the same genome s_k, and each doubly-unique substring $d_{j,i'} \in D_j$ can originate from s_k, or

ii. $U_j = \emptyset$, however the intersection between the genomes where the doubly-unique substrings of r_j can originate from is comprised of only one genome, s_k, or

iii. $U_j = \emptyset$ and d_j is comprised of doubly-unique substrings that can only originate from the same pair of genomes s_k and $s_{k'}$, then

then increase $c(u_{j,i})$ by 1 for each $u_{j,i} \in U_j$ and $c(d_{j,i})$ by 1 for each $d_{j,i} \in D_j$.

4 Proof of Theorem 1

We begin with the following theorem from Weissman et al. [5] that bounds the L1 distance between the empirical distribution of a sequence of independent, identically distributed random variables and the true distribution.

Theorem 8. *Let P be a probability distribution on the set $\mathcal{A} = \{1, \cdots, a\}$. Let $X_1, X_2, \cdots, X_n$ be i.i.d. random variables distributed according to P. Then, for any given $\epsilon > 0$,*

$$Pr[||P - \bar{P}||_1 \geq \epsilon] \leq (2^a - 2)\exp(-n\epsilon^2/2)$$

where $\bar{P}$ is the empirical estimation of P defined as $\bar{P}(i) = \frac{\sum_{j=1}^{n} \delta(X_i = j)}{n}$, where $\delta(e) = 1$ if and only if e is true and $\delta(e) = 0$ otherwise.

Now consider a collection of genomes $\mathcal{A} = \{s_1, \cdots, s_a\}$ with relative abundances $p_1, \cdots, p_a$ and the set $\mathcal{Q} = \{r_1, \cdots, r_n\}$ of n reads (i.e., L-mers) sampled independently and uniformly at random from $\mathcal{A}$ according to $p_1, \cdots, p_a$. On each genome s_i let n_i^L denote the total number of L-mers and $q_i = \frac{nu_i^L}{n_i^L}$ be the proportion of unique L-mers; then the probability of a read $r_j \in \mathcal{Q}$ corresponds to a unique L-mer on s_i is $\frac{p_i n_i^L}{\sum_{i'=1}^{a} p_{i'} n_{i'}^L} \cdot q_i$, and the probability of a read $r_j \in \mathcal{Q}$ does not correspond to any unique L-mers on s_i is $\frac{p_i n_i^L}{\sum_{i'=1}^{a} p_{i'} n_{i'}^L} \cdot (1 - q_i)$. Therefore $r_1, \cdots, r_j$ are i.i.d. distributed according to $(\frac{p_1 n_1^L}{\sum_{i'=1}^{a} p_{i'} n_{i'}^L} \cdot q_1, \cdots, \frac{p_a n_a^L}{\sum_{i'=1}^{a} p_{i'} n_{i'}^L} \cdot q_a, \sum_{i=1}^{a} \frac{p_i n_i^L}{\sum_{i'=1}^{a} p_{i'} n_{i'}^L} \cdot (1 - q_i)) = (p'_1 q_1, \cdots, p'_a q_a, \sum_{i=1}^{a} p'_i (1 - q_i))$ where the last term corresponds to the reads that are not unique to any $s_i \in \mathcal{A}$.

Proof of Theorem 1. Let c_i be the number of reads assigned to s_i. Also let $p' = (p'_1, \cdots, p'_a)$. We set $\hat{p} = (\hat{p_1}, \cdots, \hat{p_a})$, by defining $\hat{p_i} = \frac{c_i/q_i}{n}$ to be the predicted abundance of s_i based on the number of reads assigned to it. Consider $P = (p'_1 q_1, \cdots, p'_a q_a, \sum_{i=1}^{a} p'_i (1 - q_i))$ and $\hat{P} = (\frac{c_1}{n}, \cdots, \frac{c_a}{n}, 1 - \sum_{i=1}^{a} \frac{c_i}{n}) = (\hat{p_1} q_1, \cdots, \hat{p_a} q_a, 1 - \sum_{i=1}^{a} \frac{c_i}{n})$; by definition, we have $||P - \hat{P}||_1 \geq \sum_{i=1}^{a} |p'_i - \hat{p_i}| q_i \geq q_{\min} \cdot \sum_{i=1}^{a} |p'_i - \hat{p_i}| = q_{\min} \cdot ||p' - \hat{p}||_1$. Then the following three theorem statements hold.

- (i) Given that $n \geq \frac{2(a+1) + \ln \frac{1}{\zeta}}{(p_{\min} q_{\min})^2}$, we have

$$\begin{aligned}
\Pr\Big[||p' - \hat{p}||_1 \geq p_{\min}\Big] &= \Pr\Big[q_{\min}||p' - \hat{p}||_1 \geq p_{\min} q_{\min}\Big] \\
&\leq \Pr\Big[||P - \hat{P}||_1 \geq p_{\min} q_{\min}\Big] \\
&\leq 2^{a+1} \exp(-n(p_{\min} q_{\min})^2/2) \\
&\leq 2^{a+1} \exp(-\frac{2(a+1) + \ln \frac{1}{\zeta}}{(p_{\min} q_{\min})^2} (p_{\min} q_{\min})^2/2) \\
&= \frac{2^{a+1}}{e^{a+1}} \zeta \\
&\leq \zeta.
\end{aligned}$$

This implies that with probability $\geq 1 - \zeta$ the L1 distance between p' and $\hat{p}$ is upper bounded by $p_{\min}$. As a result we have $\hat{p_i} > 0$ for each $\hat{p_i}$, i.e. $c_i \geq 0$.

- (ii) The proof follows by simply replacing $p_{\min}$ with ϵ in the proof of (i).
- (iii) The proof follows by simply replacing $p_{\min}$ with $\sqrt{\frac{2(\ln\frac{1}{\zeta}+(a+1))}{nq_{\min}^2}}$ in the proof of (i). Specifically,

$$\begin{aligned}
\Pr\Bigg[||p'-\hat{p}||_1 \geq \sqrt{\frac{2(\ln\frac{1}{\zeta}+(a+1))}{nq_{\min}^2}}\Bigg] &= \Pr\Bigg[q_{\min}||p'-\hat{p}||_1 \geq q_{\min}\cdot\sqrt{\frac{2(\ln\frac{1}{\zeta}+(a+1))}{nq_{\min}^2}}\Bigg] \\
&\leq \Pr\Bigg[||P-\hat{P}||_1 \geq \sqrt{\frac{2(\ln\frac{1}{\zeta}+(a+1))}{n}}\Bigg] \\
&\leq 2^{a+1}\exp(-\frac{n}{2}\cdot\frac{2(\ln\frac{1}{\zeta}+(a+1))}{n}) \\
&= \frac{2^{a+1}}{e^{a+1}}\zeta \\
&\leq \zeta.
\end{aligned}$$

5 The Experimental Setup: Index Datasets, Queries and Benchmarked Methods

5.1 Species-Level Index Dataset

`species-level-all` **Index Dataset.** Our `species-level-all` index dataset consists of all complete archaeal, bacterial, and viral genomes in NCBI's RefSeq Database [6] (as per the "RefSeq-CG" dataset introduced in the recent benchmark [7]). For the `species-level-all` index dataset we used release version 205 of RefSeq, downloaded on 05/28/2021. We (randomly) selected one representative genome per species out of 32454 complete reference genomes representing respectively 6040, 10087 and 291 distinct bacterial, viral and archaeal species. This resulted in a total of $m = 16418$ genomes with a total length of $M = 5.5 * 10^{10}$, including the reverse complement contigs. On this index dataset we used the CAMI and IMMSA queries - as will be described below in **Supplementary Notes 5.4.1 and 5.4.2**. On these queries we benchmark the performance of `CAMMIQ` on genome identification against available metagenomics classification and profiling tools, namely, Kraken2 [8] (the latest version of Kraken [9]), KrakenUniq [10], CLARK [11], Centrifuge [12] and Bracken [13]. These five tools provide very similar functionality to `CAMMiQ` such as read level classification and abundance estimation. We built indices for `CAMMiQ`, Kraken2, KrakenUniq, CLARK, Centrifuge and Bracken all on the `species-level-all` index dataset to support the CAMI and IMMSA queries.

`species-level-bacteria` **Index Dataset.** Our `species-level-bacteria` index dataset consists of all complete bacterial genomes in an earlier release version of RefSeq, i.e., release version 93, downloaded on 06/16/2019. Again, we (randomly) selected one representative genome per species out of 13737 complete reference genomes representing 4122 distinct species. This resulted in a total of $m = 4122$ genomes with a total length of $M = 3.4 * 10^{10}$ including the reverse complement contigs. On this index dataset we composed 14 queries - including 10 challenging queries and 4 easier queries - as will be described in **Supplementary Note 5.4.2**. On these queries we benchmarked `CAMMiQ` against Kraken2, KrakenUniq, CLARK, Centrifuge, Bracken, and additionally against MetaPhlAn2, a marker-gene based profiling tool, with an emphasis on genome identification and quantification (profiling). We built indices for `CAMMiQ`, Kraken2, KrakenUniq, CLARK, Centrifuge and Bracken on the `species-level-bacteria` index dataset. For MetaPhlAn2 we used its own (prebuilt) index, as it does not support indices built on a custom dataset.

5.2 Strain-Level Index Dataset

Our `strain-level` index dataset is smaller: it is restricted to 614 Human Gut related genomes/strains (according to [14]) in RefSeq[1] and is designed to evaluate `CAMMiQ`'s strain-level identification and quantification

[1]The complete set of genomes in this database is 617 but only 614 can be downloaded from RefSeq.

performance against the available tools mentioned above. For the `strain-level` index dataset we used release version 93 of RefSeq. Majority of these genomes are incomplete and are comprised of multiple contigs; we filtered out any contig with length < 10KB and built the index on the remaining contigs. This resulted in seven strains without a single unique 100-mer and one strain without a single unique or doubly-unique 100-mer. The last genome with no unique or doubly-unique 100-mers, *Bacillus andreraoultii*, was excluded when generating our `strain-level` query sets since it contains no indexable substring. We simulated 4 query sets for this index dataset, see **Supplementary Note 5.5** for the detailed information of these query sets. Finally, on the `strain-level` index dataset we built indices for `CAMMiQ`, Kraken2, KrakenUniq, Centrifuge and CLARK (we did not report Bracken results as it was not designed to handle strain level queries), with the same setting as `species-level-bacteria` index dataset. For MetaPhlAn2, we used its own (prebuilt) index, as it does not support indices built on a custom dataset.

5.3 Subspecies-Level Index Dataset

Perhaps our most interesting results are on the `subspecies-level` index dataset, which consists of 3395 selected genomes from the 13737 complete bacterial genomes in release version 93 of RefSeq; this dataset was primarily designed to evaluate `CAMMiQ`'s accuracy on real metatranscriptomic query sets obtained from 262 single human immune cells [15], each exposed to or infected with distinct *Salmonella* strains.

To create our `subspecies-level` dataset, we first identified 2753 out of the total 4122 species to which at least 10 reads were mapped using GATK PathSeq [16] - and then we subsampled the 4325 (out of the total 13737) genomes from these 2753 species by only keeping one representative for each child of a species level taxonomic ID in NCBI's Taxonomy Database; The only exception is we replaced the sampled genome of *Salmonella enterica subsp. enterica* (Taxonomy ID: 59201) with the genome of the two related strains *D23580* (Taxonomy ID: 568708) and *LT2* (Taxonomy ID: 99287), which results in the final 3395 subspecies level representatives. As a final step, we removed all plasmids contained in these genomes, since the plasmids in many genomes downloaded from RefSeq were missing, which may cause potential false positive "unique" or "doubly unique" substrings. On these 3395 genomes we built the index for `CAMMiQ` and PathSeq to support the scRNA-seq queries (see **Supplementary Note 5.6**).

5.4 Species-Level Queries

The species-level queries consist of (i) 8 CAMI query sets and 8 IMMSA query sets utilized in two recent benchmark studies for comparing metagenomic profiling tools [7, 17], on which we ran our $\mathcal{A}_2$ type of query; and (ii) 14 synthetic query sets constructed by sampling reads uniformly at random from selected genomes in our `species-level-bacteria` index dataset (as such referred to as `species-level-bacteria` queries/query sets), on which we ran our $\mathcal{A}_3$ type of query. Both query sets are summarized in **Table 1** in the main text.

5.4.1 IMMSA Query Sets

The 8 IMMSA query sets, namely "IMMSA-buccal-12", "IMMSA-citypark-48", "IMMSA-gut-20", "IMMSA-house-31", "IMMSA-house-21", "IMMSA-soil-50", "IMMSA-simBA-525" and "IMMSA-nycsm-20" were initially compiled in [17] to standardize the benchmark of metagenomic classification and profiling tools (see `https://www.microbialstandards.org/standards-related-resources`), and later adopted in another leading benchmark [7] of metagenomics tools. All of these 8 query sets are synthetic - the first 6 queries were created using the ART simulator with default error and quality base profiles [18] to simulate 100-bp Illumina reads from selected sets of reference genomes. Each of these 6 query sets represents a distinct microbial habitat based on studies that characterized real metagenomes found in the human body (mouth, gut, etc.) and in the natural or built environment (city parks/medians, houses, and soil). The seventh query set, "IMMSA-simBA-525" comprised 100-bp simulated reads from 525 randomly selected species. The last query set, "IMMSA-nycsm-20", was created to represent the organisms of the New York City subway system as described in the study of Afshinnekoo et al. [19], using the same methodology as in [20]. Together, these eight unambiguously mapped query sets of reads contain a total of 657 distinct species.

Note that reads in the 8 IMMSA benchmark query sets were not sampled from any (including the most

Query Set	Num. Species	Num. Species in Index Dataset	Num. Reads	Num. Reads in Index Dataset
IMMSA-buccal-12	12	11	600000	550000
IMMSA-citypark-48	48	47	1200000	1175000
IMMSA-gut-20	20	19	500000	475000
IMMSA-house-30	30	27	750000	660185
IMMSA-house-20	20	19	500000	475000
IMMSA-soil-50	50	48	2500000	2400000
IMMSA-simBA-525	525	492	5727654	5402857
IMMSA-nycsm-20	20	19	500000	475000

Supplementary Table 1: Additional information on the 8 IMMSA query sets. The second and fourth column indicate the total number of species and number of reads in each query set, respectively. The third and fifth column indicate the number of species and the number of reads from a genome in the `species-level-all` index dataset for each query set, respectively.

Query Set	Num. Species	Num. Species in Index Dataset	Num. Reads
CAMI-LC-1	23	4	99796358
CAMI-MC-1	72	22	99837678
CAMI-MC-2	72	22	99787568
CAMI-HC-1	243	20	99811870
CAMI-HC-2	243	20	99808454
CAMI-HC-3	243	20	99809214
CAMI-HC-4	243	20	99805006
CAMI-HC-5	243	20	99803592

Supplementary Table 2: Additional information on the 8 CAMI query sets. The second and fourth column indicate the total number of species and number of reads in each query set, respectively. The third column indicates the number of species (whose taxonomic ID is) included in the `species-level-all` index dataset, for each query set.

comprehensive `species-level-all`) index dataset[2]. However, to measure the classification performance, namely precision and recall, we still obtained the "ground truth" genome for each read r_j by first mapping the contig accession number that produces r_j to a species level taxonomic ID (through querying NCBI accession2taxid database `https://ftp.ncbi.nlm.nih.gov/pub/taxonomy/accession2taxid/`), and then pick the genome within that taxonomic ID (as we only kept one representative genome per species in our `species-level-all` index dataset). We consider the ground truth of any reads that were not assigned to a genome in our `species-level-all` index dataset as "unassigned" - any assignment by `CAMMiQ` or other tools built on such index dataset was considered "false positive". In addition, to measure the performance of species level identification, we consider the true number of species existing in each of the 8 query sets as the number of genomes included in the `species-level-all` index dataset (i.e., having at least one read assigned to due to the procedure above), regardless of how many species these query sets of reads were initially sampled from. See **Supplementary Table 1** for more details.

5.4.2 CAMI Query Sets

The CAMI query sets include 1 low complexity (in terms of number of species in the mixture) metagenome containing bacteria, and viruses, namely 'CAMI-LC-1'; 2 medium complexity metagenomes containing archaea, bacteria and viruses, namely 'CAMI-MC-1' and 'CAMI-MC-2'; and 5 high complexity metagenomes containing archaea and bacteria, namely 'CAMI-HC-1' to 'CAMI-HC-5'. These metagenomes contain only

[2]In fact, reads may be sampled from multiple incompletely assembled genomes or plasmids from the same species. As such, we run the $\mathcal{A}_2$ type of query for `CAMMiQ` on these query sets.

about 30-40% of genomes from known species in these kingdoms, and as such even less genomes in the index dataset, see **Supplementary Table 2**. The rest of the abundance in these samples is characterized by (i) added plasmids, (ii) novel new species, genera, and (iii) simulated evolved strains from existing species. Reads in these queries were simulated as 150bp mate-pairs with insert size 270bp. Note that the ground truth on these queries only include the abundance of each species (and higher taxonomies), but not the species (or lower) level taxonomic ID for each individual read. Therefore, we focus on the species level genome identification performance, but not on read classification performance. We assume a true positive genome if its **species level taxonomic ID exists in the ground truth files**, even if that genome may not have been used in the CAMI simulation process.

5.4.3 `species-level-bacteria` Query Sets

We generated 14 query sets based on `species-level-bacteria` index dataset, including 10 challenging queries and 4 easier queries. Details of these query sets are summarized in **Supplementary Table 3**.

Challenging Queries. The first set of simulated metagenomes aim to assess how well `CAMMiQ` identifies species in a query. For that we simulated a metagenome consisting of the 20 genomes that have the lowest number of unique L-mers in our species-level dataset. Each genome in the mixture was simulated to have the same read coverage (indicated by **uniform** in the name of a query). The very first query we generated from this mixture, Least-20-uniform-1, has no read errors. The second query, Least-20-uniform-2, comes with i.i.d. substitution errors occurring at a rate of 1%. The third query, Least-20-uniform-3, is distinct to the previous two queries in the way that reads are sampled with a GC bias (See "GC Bias" below) applying to each of the 20 genomes. Additionally, Least-20-uniform-3 also comes with 1% error rate. Note that the 20 genomes we used in this mixture are intrinsically difficult to be identified by CLARK and other tools we compared. However since these genomes have many doubly-unique L-mers, which are sometimes shared with multiple other genomes, they could be identified by `CAMMiQ` (see **Supplementary Figure 2** for a more detailed explanation).

The second set of metagenomes we simulated aim to assess the species-level quantification performance of `CAMMiQ`. This simulated metagenome consists of the 20 genomes from among the 50 genomes in our species-level dataset with the lowest proportion of unique L-mers, but has the highest proportion of doubly-unique L-mers, making them somewhat easier to identify in comparison to the simulated metagenome above, but difficult to quantify by tools other than `CAMMiQ`. We again synthesized three queries from this mixture, with no sequencing errors in the first query Least-quantifiable-20-uniform-1; with 1% substitution error in the second query Least-quantifiable-20-uniform-2; and with GC bias as well as 1% error in the last query Least-quantifiable-20-uniform-3. The ability of `CAMMiQ`'s ILP formulation to simultaneously determine the presence and abundance of genomes in these queries help it outperform the alternatives. (See also **Supplementary Figure 2** for a more detailed explanation.)

Supplementary Figure 2 presents 50 genomes in our species-level dataset with the least number of unique L-mers. In this graph each node represents one such genome; each edge connects two nodes if they share a doubly-unique substring. Solid black edges indicate a pair of nodes that share at least 30 doubly-unique substrings; the remaining edges in grey indicate node pairs with fewer number of shared doubly-unique substrings. Notice that there is a special node in the center, representing the union of all genomes not included in these 50-genome subset. Any node connected to this special node by a single edge, or by a path, is identifiable and quantifiable by `CAMMiQ`, provided that all edges in this path are black (22 of these 50 nodes are as such) or all nodes in this path have sufficient abundance. Note that 20 of the genomes here are connected to this special node by a black edge: these are the genomes that form the 3 least-quantifiable-20 queries in our experiments.

Even though RefSeq identifies each genome in our species-level dataset to represent a distinct species, a few of them have "unclassified" lineages at the species level. (Some of the genomes in the above queries are among them; see **Supplementary Figure 2**.) For example, *Rhizobium sp. N1314* with Taxonomy ID: 1703961 has Rank: species; however its Lineage is noted as *unclassified Rhizobium.* Because of this ambiguity, we generated a third set of metagenomes, again consisting 20 of those 50 genomes with the lowest proportion of unique L-mers, this time making sure that each of these 20 genomes represent a distinct genus. We synthesized 4 queries from this set of genomes. The first one has the same read coverage over all genomes,

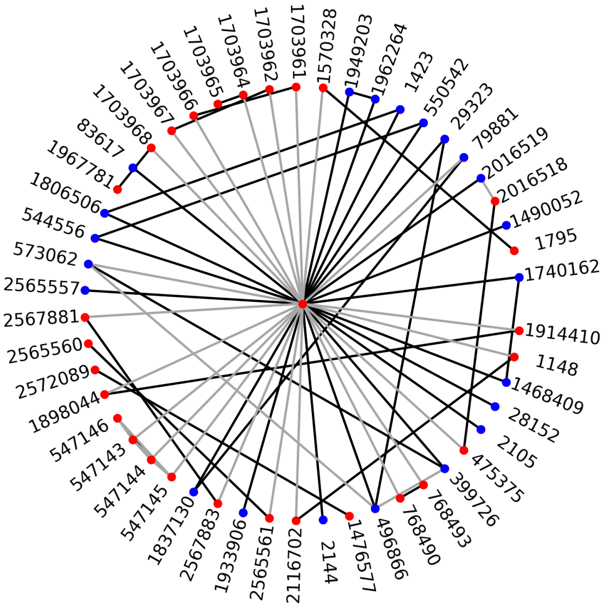

Supplementary Figure 2: Shared doubly-unique substrings among the 50 genomes with the least number of unique L-mers in our species-level dataset consisting of 4122 RefSeq bacteria genomes. Each node represents one of these 50 genomes, labeled with its NCBI taxID at species level. The central node specially represents the remaining 4072 genomes. An edge connecting two nodes indicate at least one doubly-unique substring shared between them. A black edge indicates ≥ 30 doubly-unique substrings in `CAMMiQ`'s index shared between the two corresponding genomes. All other edges in grey imply < 30 shared doubly-unique substrings. A blue-colored node indicates one that is connected to the central node through a path of black edges. As such, they are relatively easy to identify and quantify; 22 of these 50 nodes are blue. The remaining (red) nodes can be identified by `CAMMiQ` provided they have "sufficient abundance" in the query.

and no GC bias as well as sequencing errors, denoted as Least-20-genera-uniform-1. The second query has the same setup, except a 1% substitution errors. The third query has 1% substitution errors and also GC bias. Finally, the coverage of each genome in the last query (denoted Least-20-genera-lognormal) follows a lognormal distribution with mean 0.0 and standard deviation 1.0.

Easier Queries. In addition to the above particularly challenging queries, we simulated a number of additional read collections from 20 to 100 randomly chosen genomes from our species-level dataset. All these read collections come with i.i.d. substitution errors; the first three queries at a rate of 1% and the last query at a rate of 0.6%. Among them, the first simulated query (denoted Random-20-uniform) included reads from 20 genomes, each with similar read coverage. The second (denoted Random-20-lognormal) again included reads from 20 genomes, this time with coverages following a log-normal distribution with mean 0.0 and standard deviation 1.0. The third (denoted Random-20-lognormal-a.g.) included reads from 20 genomes, again with log-normal coverage distribution; what makes this query unique is that 10% of the reads were from an additional genome (denoted in the dataset name as a.g.) not included in our species-level dataset and thus is not part of `CAMMiQ`'s index. The fourth (denoted Random-100-uniform) included reads from 100 randomly chosen genomes from our species-level dataset, all with the same coverage.

GC Bias. To demonstrate the impact of non-uniform coverage from metagenomic datasets to our $\mathcal{A}_3$ type queries, we simulated 3 challenging query sets with GC bias from the `species-level-bacteria` index dataset; namely Least-20-uniform-3, Least-quantifiable-20-uniform-3, and Least-genera-20-uniform-3. We applied the following GC bias model used in a recent Illumina reads simulator InSilicoSeq [21]: randomly pick one read from the genome; if the proportion of GC is between 0.4-0.6, we always keep the read; if the proportion is between 0.2-0.4 or 0.6-0.8, we keep the read with probability 0.5; if the proportion is between

Query Set	GC Bias	Dist.	Error Rate	Num. Genomes	Num. Reads
Least-20-uniform-1	N	Uniform	0	20	4.8M
Least-20-uniform-2	N	Uniform	0.01	20	4.8M
Least-20-uniform-3	Y	Uniform	0.01	20	4.8M
Least-quantifiable-20-uniform-1	N	Uniform	0	20	5.0M
Least-quantifiable-20-uniform-2	N	Uniform	0.01	20	5.0M
Least-quantifiable-20-uniform-3	Y	Uniform	0.01	20	5.0M
Least-20-genera-uniform-1	N	Uniform	0	20	4.0M
Least-20-genera-uniform-2	N	Uniform	0.01	20	4.0M
Least-20-genera-uniform-3	Y	Uniform	0.01	20	4.0M
Least-20-genera-lognormal	N	Lognormal	0.01	20	4.0M
Random-20-uniform	N	Uniform	0.01	20	4.4M
Random-20-lognormal	N	Lognormal	0.01	20	5.0M
Random-20-lognormal-a.g.	N	Lognormal	0.01	21	1.1M
Random-100-uniform	N	Uniform	0.006	100	21.5M

Supplementary Table 3: Additional information on the 14 `species-level-bacteria` query sets, including (i) whether GC bias was introduced through simulation; (ii) distribution of genomes in the mixture; (ii) error rate; (iv) number of genomes in each query set; and finally (v) number of reads in each query set.

Query Set	Min P-value	Max P-value	Median P-value
Least-20-uniform-3	$5.084 * 10^{-7}$	0.7406	0.0004
Least-quantifiable-20-uniform-3	$2.976 * 10^{-14}$	0.1162	0.0002
Least-20-genera-uniform-3	$2.880 * 10^{-9}$	0.3212	0.0024

Supplementary Table 4: The 3 `species-level-bacteria` query sets with GC bias. Min/Max/Median P-value: the smallest (most significant), largest (least significant) and median P value, with two sample Kolmogorov-Smirnov tests, of uniform read sampling on a single genome, among the 20 genomes in each query set.

0-0.2 or 0.8-1.0, we keep the read with probability 0.1. We repeated the process until sufficient (i.e., the same as the corresponding queries without GC bias) number of reads were sampled on each genome. Note that the distribution of reads across different genomes in the mixture is still uniform in all 3 cases.

We then compared the distributions of reads on each genome with and without GC bias procedure, through a two-sample Kolmogorov-Smirnov (KS) test. For each genome, we obtained the distribution of the number of reads in each 25Kbp bin both with and without GC-bias on that genome. We conducted the two-sample KS test for each of the 20 genomes in each query set. As demonstrated in **Supplementary Table 4**, most genomes showed a significant difference between uniform and GC-bias (non-uniform) sampling. We will demonstrate, however, such significant GC-bias has only limited impact on the $\mathcal{A}_3$ (genome quantification) queries in main text, **Table 3**.

5.5 Strain-Level Queries

To assess `CAMMiQ`'s performance in strain level identification and quantification, we also simulated 4 queries involving genomes from a database of 614 genomes of human gastrointestinal bacteria [14] from 409 species and 515 strains. The first one involving reads from 25 strains with the smallest number of unique L-mers (denoted HumanGut-least-25), next two involving reads from randomly selected 100 strains, the first with $L = 100$ as per the remainder of the queries (denoted HumanGut-random-100-1), and the second with $L = 125$ (denoted HumanGut-random-100-2), and the final involving reads sampled from 409 randomly picked strain level genomes (denoted HumanGut-all), each from a distinct species (species-level taxonomic ID) in the index dataset - which made it easier for us to determine the total number of genomes identified by other tools like Kraken2 etc. in a query. Note that we additionally excluded the genomes with neither unique nor doubly-unique 100-mers when sampling the genomes to compose our `strain-level` queries. The

4 `strain-level` queries are also summarized in **Table 1**, and on these queries, we evaluated `CAMMiQ` on the most general $\mathcal{A}_3$ type queries.

5.6 Subspecies-Level Queries

In our final experiment, we applied `CAMMiQ` to a "gold-standard" query set consisting of immune cells infected *ex vivo* with an *intracellular* bacteria *Salmonella enterica* and subsequently sequenced using single-cell RNAseq (scRNA-seq) [15] to validate its feasibility of identifying microbial reads from real datasets. Specifically, this query, denoted as Filtered-scRNA-seq and also summarized in **Table 1**, consists of 262 monocyte-derived dendritic cells (moDCs) infected with either the *D23580* (STM-D23580) or the *LT2* (STM-LT2) strain of *Salmonella enterica* and sequenced using Smart-seq2 platform. Additionally there are 80 uninfected cells used as negative controls. The reads from each cell (out of the total 342 cells) forms a natural query set for this index dataset. We preprocess the queries to filter out the reads potentially originate from human genome by aligning the reads to the reference human genome using STAR aligner [22]. Then, we remove all mapped reads and use the remaining as our scRNA-seq queries. A recent study [23] used the GATK PathSeq tool [16] to validate the *Salmonella* strains associated with each cell with limited success. Unlike the tools benchmarked above, PathSeq is alignment-based; as a consequence it is slower than the above alternatives but is expected to be more accurate.

The corresponding index we built to respond to this query consisted of the sparsified set of unique and doubly-unique substring (with $L = 75$, $L_{\min} = 26$ and $L_{\max} = 50$) from our `subspecies-level` dataset of 3395 selected complete bacterial genomes in NCBI's RefSeq Database. Interestingly, `CAMMiQ`'s ability to distinguish cells exposed to or infected with specific strains of *Salmonella* was better than PathSeq's ability to do the same (importantly, with the same index dataset) - with the added bonus that it is much faster. Also note that on metatranscriptomic query sets we used query types $\mathcal{A}_1$ and $\mathcal{A}_2$, rather than the most general query type $\mathcal{A}_3$.

6 Setup and Parameters Used for Individual Software Tools in Our Benchmarking Study

6.1 Accounting for Genome Lengths in Abundance Profiles

A number of tools, including `CAMMiQ`, Centrifuge, Bracken and MetaPhlAn2, output *read depth* (a.k.a. *read coverage*) as "abundance"; i.e., they report as abundance the number of reads assigned to a certain genome, normalized by genome length. Other tools, including Kraken2, KrakenUniq and CLARK, simply report the total number of reads assigned to each genome (or each taxonomic rank) as its abundance. Throughout this paper we compute the L1 or L2 distance between the estimated abundance and true abundance using the normalized abundance values for `CAMMiQ`, Bracken and MetaPhlAn2:

$$Lp_{\{\texttt{CAMMiQ}, \text{ Bracken, Centrifuge, MetaPhlAn2}\}} = \sum_{i=1}^{a} |p_i - \hat{p_i}|^p, (p = 1, 2),$$

where p_i is the (normalized) read depth for genome s_i in the ground truth $\mathcal{A} = \{s_1, \cdots, s_a\}$; $\hat{p_i}$ is the abundance of genome s_i reported by each individual software tool. On the other hand, we use total read counts as abundance values for Kraken2, KrakenUniq and CLARK:

$$Lp_{\{\text{Kraken2, KrakenUniq, CLARK}\}} = \sum_{i=1}^{a} |p'_i - \hat{p_i}|^p, (p = 1, 2),$$

where p'_i is the (normalized) total read count for genome s_i in the ground truth $\mathcal{A} = \{s_1, \cdots, s_a\}$, which can be obtained by multiplying the read depth by genome length and then renormalized by the sum over all genomes in the ground truth

$$p'_i = \frac{p_i \cdot |s_i|}{\sum_{i'} p_{i'} \cdot |s_{i'}|};$$

and $\hat{p_i}$ is again the abundance of genome s_i reported by each individual software tool.

6.2 `CAMMiQ` Commands and Setup

Index construction.

`cammiq --build --both -k 26 -L 100 -Lmax 50 -h 26 -f genome_map.out -D cammiqDB -i index_u.bin1 index_d.bin2 -t 32`

Unless otherwise specified (see **Supplementary Note 8**), we required (i) minimum substring length (`-k`) 26 for both unique and doubly-unique substrings; (ii) maximum substring length (`-Lmax`) 50 for both unique and doubly-unique substrings; and (iii) read length (`-L`) 100 in `CAMMiQ` **index construction**, even if some query sets (e.g. the 8 CAMI queries) have reads longer than 100. Note that (unlike Bracken, which will be described later,) `CAMMiQ` indices constructed with `-L 100` can naturally support querying reads longer than 100. We assume all genomes (`*.fna`) are stored under the directory `cammiqDB`. The required format of CAMMiQ `genome_map.out` file can be found at `https://github.com/algo-cancer/CAMMiQ`.

Query.
(1) Compute $\mathcal{A}_1$. `cammiq --query --read_cnts -h 26 26 -f genome_map.out -i index_u.bin1 index_d.bin2 -q query.fq -o cammiq.out -t 1`

(2) Compute $\mathcal{A}_2$. `cammiq --query --read_cnts --doubly_unique -h 26 26 -f genome_map.out -i index_u.bin1 index_d.bin2 -q query.fq -o cammiq.out -t 1`

(3) Compute $\mathcal{A}_3$. `cammiq --query -h 26 26 -f genome_map.out -i index_u.bin1 index_d.bin2 -q query.fq -o cammiq.abundance -t 1`

CAMMiQ query requires a *single-end* fastq `query.fq` as input, and output (i) the set of genomes in $\mathcal{A}_1$, as well as the number of reads assigned uniquely to each genome, when computing $\mathcal{A}_1$; (ii) the minimum set of genomes $\mathcal{A}_2$, as well as the number of reads assigned uniquely or doubly-uniquely to each genome when computing $\mathcal{A}_2$; (iii) the set of genomes in $\mathcal{A}_3$ along with the abundance of each genome, when computing and $\mathcal{A}_3$. Note that the $\mathcal{A}_1$ and $\mathcal{A}_2$ collections can be further processed by keeping only genomes with a sufficient counts of unique (and/or doubly-unique) substrings covered by reads in the query (See below and **Section Query Processing Stage 1: Preprocessing the Reads**, main text).

Note additionally that `CAMMiQ`'s primary goal is not to classify each read, but rather to identify and quantify genomes in a query. Nonetheless, we still report its intermediate output as follows. `CAMMiQ` considers certain reads as conflicting; here we consider them as not assigned. It assigns certain reads to a single genome; we consider each such read assigned, and if the assignment is correct, also correctly assigned. `CAMMiQ` then assigns each remaining read ambiguously to two potential genomes.[3] We consider this read assigned when evaluating its classification precision and recall with our species level queries (i.e., the 16 CAMI and IMMSA queries and 14 `species-level-bacteria` queries), and in case one of these two genomes is correct, also correctly assigned.

Unless otherwise specified, we ran `CAMMiQ` with $\alpha = 0.0001$ for $\mathcal{A}_3$ and similarly by filtering out genomes with unique or doubly-unique counters add up to a value smaller than $0.0001nu_i^L$ or $0.0001nd_i^L$ respectively. For `subspecies-level` (scRNA-seq) queries, we alternatively set up a hard threshold t to filter out genomes with unique or doubly-unique counters add up less than t, see **Figure 2b** in the main text.

6.3 MetaPhlAn2 Commands and Setup

Query.

`python metaphlan2.py query.fq --input_type fastq -o abundance.txt --nproc 1`

We also ran MetaPhlAn2 with a *single-end* fastq `query.fq` as input, and used the abundance values re-

[3]This happens if the read includes one or more doubly-unique substrings from the same pair of genomes but no unique substring.

ported in its default output, `abundance.txt`, to compare with other tools. Note that the default abundance cut-off for MetaPhlAn2 is 0.01%, i.e., MetaPhlAn2 does not report any genome with abundance less than 0.01% of the total abundance in `abundance.txt`.

For MetaPhlAn2, we did not give the precision and recall values in `species-level-bacteria` queries. This is primarily due to MetaPhlAn2's use of an index based on a predetermined and very different database of marker genes, which contains insufficient information to correspond them to the taxonomic IDs of the genomes indexed by other software tools. As such, we could not claim a read assignment by MetaPhlAn2 is "correct" and measure the precision and recall for read classification. In fact, the goal of MetaPhlAn2 is not to assign reads to genomes, but to identify distinct genomes in a metagenomic sample (as per `CAMMiQ`); so it is less meaningful to consider its classification performance. It was deigned as an alignment based tool - and to reduce the intensive alignment task, MetaPhlAn2 relies on a much smaller database of marker genes, which is sufficient for genome identification, than the collection of (complete) genomes indexed by the other four tools. As a consequence, it only assigns very few reads to the marker genes in its database and thus would have very low recall (even if all reads were assigned correctly) as discussed in the main text.

As MetaPhlAn2 does not explicitly support taxonomic IDs, we have to map the name of each item in its output at a given taxonomic level (genus or species level) to the corresponding taxonomic ID. In **Table 3** and **Supplementary Table 5**, we mapped each **species level** output from MetaPhlAn2 to a **genus level** taxonomic ID, and then (i) computed the F1 scores by assuming all genomes at species level within some genus g in a query were correctly identified, if the taxonomic ID of g is found in post-processed MetaPhlAn2 output; and (ii) computed the L1 and L2 error by summing up the difference between the true abundance of each genus g and the sum of the predicted abundance for all species map to g. When measuring its strain level performance in **Table 5**, we mapped each **strain level** output from MetaPhlAn2 to a **species level** taxonomic ID, and computed the performance metrics in a similar way. However, such mapping at strain level is not guaranteed to be correct.

6.4 Kraken2 Commands and Setup

Index construction.
(1) Install taxonomy tree

```
kraken2-build --download-taxonomy --db KrakenDB
```

(2) Prepare all genomes in the index dataset in Kraken2 format. Note that this step was repeated for all fasta files and the running time for this step was included in Kraken2's total running time.

```
kraken2-build --add-to-library genome.fna --db KrakenDB
```

(3) Build index with 32 threads, with k-mer length 26.

```
kraken2-build --build --db KrakenDB --threads 32 --kmer-len 26 --minimizer-len 21
--minimizer-spaces 5
```

Query.

```
kraken2 --db KrakenDB --output query.reads --report-file query.report --threads 1
query.fq
```

Each Kraken2 query takes a *single-end* fastq `query.fq` as input, and output 2 files including `query.reads`, which specifies the assignment of each read; and `query.report`, which specifies the abundance of each taxonomic rank. We measured read classification performance by parsing `query.reads`, and genome identification/quantification performance by parsing `query.report`. We always forced a single thread for comparing the query time for each tool we tested. All other parameters were default.

Kraken2 usually takes a minimal amount of time to preload its index in to memory. Even if that is the case, we still exclude its index loading time when reporting Kraken2 query time in **Table 4**, main text.

6.5 KrakenUniq Commands and Setup

Index construction.
(1) Download taxonomy tree

```
krakenuniq-download --db KrakenUniqDB taxonomy
```

(2) Create a subdirectory named '`library`' and copy all genomes (`*.fna`) to `library`. For each genome, we created a 3 column, tab-separated '`*.map`' file also under the `library` directory, which maps each contig (accession number) to the corresponding taxonomic ID and genome (species or strain) name. As this step took minor time and was not implemented by KrakenUniq, we did not include it in KrakenUniq index construction time in **Table 4**, main text.

(3) Build the index with 32 threads, with k-mer length 26.

```
krakenuniq-build --db KrakenUniqDB --taxids-for-genomes --taxids-for-sequences --kmer-len
26
```

Query. `krakenuniq --db KrakenUniqDB -preload --threads 8`

Before running KrakenUniq queries, its index needs to be preloaded into memory (RAM). This process usually takes a couple minutes, depending on the size of KrakenUniq index. As per `CAMMiQ` and other tools, we did not measure the time for loading KrakenUniq index into memory.

```
krakenuniq --db KrakenUniqDB --fastq-input query.fq --output query.reads --report-file
query.report --threads 1
```

Each KrakenUniq query takes a single-end fastq `query.fq` as input, and output 2 files in similar format to Kraken2, including `query.reads`, which specifies the assignment of each read; and `query.report`, which specifies the abundance of each taxonomic rank. We measured read classification performance by parsing `query.reads`, and genome identification/quantification performance by parsing `query.report`. We always forced a single thread for comparing the query time for each tool we tested. All other parameters were default.

6.6 Bracken Commands and Setup

Index construction.

```
bracken-build -d KrakenDB -t 32 -k 26 -l <100/150> -x KrakenDIR
```

Bracken index construction requires the availability of (i) Kraken(2) database (including the taxonomy tree and fasta preparations in Kraken(2) format) and (ii) Kraken(2) executables. We first ran the steps (1) and (2) in Kraken2 index construction, and then input the path to `KrakenDB` in its '`-d`' option, and the Kraken2 directory, `KrakenDIR`, in its '`-x`' option.

Bracken, similar to `CAMMiQ`, requires a potential read length value (`-l`) for its index construction. To maximize its performance, we built two separate indices on our `species-level-all` index dataset, one with option `-l 150` and used for CAMI queries (read length 150); the other with option `-l 100` and used for IMMSA queries (read length 100). Recall in **Supplementary Note 6.2** that for `CAMMiQ` we only built one index on the `species-level-all` dataset with `-L 100`, and used it on both IMMSA and CAMI queries.

Query.

```
bracken -d KrakenDB -i query.report -o query.bracken -r <100/150> -l S
```

Unlike other tools which take a fastq file, a Bracken query parses and post-processes the corresponding

Kraken2 report file `query.report` and produces a new report file in similar format `query.bracken`. We measured the genome identification and quantification performance by parsing `query.bracken`. All other parameters were default. Note that Bracken can support at most species level resolution (`-l 'S'`). As such we did not run Bracken on our `strain-level` index dataset.

Bracken usually takes a minimal amount of time for both index construction and query. As it does not actually process the fastq files, we did not benchmark its running time against other tools.

6.7 Centrifuge Commands and Setup

Index construction.

```
python centrifuge-build --threads 32 --conversion-table centrifuge.conv --taxonomy-tree
nodes.dmp --name-table names.dmp --kmer-count 26 centrifuge_genomes.fna centrifugeDB
```

where `centrifuge.conv` is a two column, tab-separated file, which maps each contig (here we used the accession number for each contig as per KrakenUniq) to the corresponding taxonomic ID; `centrifuge_genomes.fna` is a combined fasta file of **all** input genomes; `centrifugeDB` is the path to the directory holding centrifuge indices. We used k-mer size 26 (the same as other tools) to construct any centrifuge indices. The taxonomy tree (`nodes.dmp` and `names.dmp`) was obtained by running step (1) for Kraken2 index construction. The running time for combining the `*.fna` input and preparing `centrifuge.conv` were excluded for Centrifuge index construction time in **Table 4**, main text.

Query.

```
centrifuge -k 1 -x centrifugeDB -U query.fq -S query.reads --report-file query.report
--threads 1
```

In centrifuge queries, we only focused on a single (i.e., the primary) assignment for each read by setting `-k 1`. We performed centrifuge queries in its single-end (-U) mode. Similar to Kraken* tools, Centrifuge outputs 2 files for each `query.fq`, including `query.reads`, which specifies the assignment of each read; and `query.report`, which specifies the abundance of each taxonomic rank. We again measured read classification performance by parsing `query.reads`, and genome identification/quantification performance by parsing query.report. We always forced a single thread for comparing the query time for each tool we tested. All other parameters were default.

6.8 CLARK Commands and Setup

Index construction and query.

```
CLARK -k 26 -T genome_map_clark.out -D CLARK_DB -O query.fq -R query -n <32/1>
```

Differing from the above tools, CLARK runs index construction and query together. It first tries to search if an index already exists under `CLARK_DB` directory. If not found, then it will first build and index and stored the index under `CLARK_DB`; otherwise it proceeds with the query. We ran CLARK commands two times. For the first time we ran CLARK with a dummy query with a single read, and measured the elapsed time for index construction. Although we used `-n 32` in this step, it turns out CLARK actually only supported a single thread during its index constriction process. Therefore, CLARK index construction time reported in **Table 4**, main text is much larger than that from other tools. For the second time, as CLARK index was already in `CLARK_DB`, we ran CLARK with `-n 1` and measured its query time. Note that we used CLARK reported query time in its log for a fair comparison to other tools, which already excluded the time to load its index into main memory/RAM.

Similar to `CAMMiQ`, CLARK queries also take a genome map file. We produced such file by keeping the `*.fna` file name and the corresponding (species or strain level) taxonomic ID for that fasta, i.e., the first and third column respectively from `genome_map.out` for `CAMMiQ`. CLARK results were stored in CSV format in

the file `query.csv` (the extension ".csv" is automatically added to the filename prefix `query` specified in `-R`). We parsed this file to measure both CLARK's read classification performance and its genome identification and quantification performance. All other parameters were default.

Query Set	`CAMMiQ`	Kraken2	KrakenUniq	CLARK	Centrifuge	Bracken	MetaPhlAn2
IMMSA-buccal-12	0.2785	0.0120	0.1782	0.1897	0.1982	0.0150	
IMMSA-citypark-48	0.5935	0.3007	0.4632	0.4360	0.4973	0.2115	
IMMSA-gut-20	0.2500	0.0174	0.1974	0.1977	0.2156	0.0278	
IMMSA-house-30	0.4685	0.0525	0.3288	0.3034	0.3768	0.0644	
IMMSA-house-20	0.3953	0.0427	0.2885	0.2810	0.3119	0.0463	
IMMSA-soil-50	0.4920	0.2953	0.4369	0.4292	0.4608	0.2201	
IMMSA-simBA-525	0.8852	0.6732	0.8756	0.8764	0.8779	0.7027	N/A
IMMSA-nycsm-20	0.2615	0.0253	0.1948	0.1789	0.2012	0.0497	
CAMI-LC-1	0.0188	0.0021	0.0076	0.0073	0.0065	0.0019	
CAMI-MC-1	0.0502	0.0112	0.0374	0.0353	0.0317	0.0108	
CAMI-MC-2	0.0694	0.0116	0.0517	0.0477	0.0409	0.0111	
CAMI-HC-1	0.0278	0.0089	0.0229	0.0231	0.0206	0.0098	
CAMI-HC-2	0.0272	0.0089	0.0225	0.0215	0.0212	0.0092	
CAMI-HC-3	0.0277	0.0088	0.0233	0.0226	0.0213	0.0097	
CAMI-HC-4	0.0268	0.0088	0.0230	0.0222	0.0208	0.0092	
CAMI-HC-5	0.0279	0.0089	0.0240	0.0231	0.0216	0.0099	
Least-20-uniform-1	1.0	0.0195	0.9189	0.9474	0.9474	0.5000	<0.7879
Least-20-uniform-2	0.8333	0.0059	0.1695	0.0255	0.2615	0.2683	<0.7879
Least-20-uniform-3	0.8163	0.0061	0.1477	0.0244	0.2481	0.3038	<0.7879
Least-quantifiable-20-uniform-1	1.0	0.0171	0.9744	1.0	0.9744	0.4615	<0.9473
Least-quantifiable-20-uniform-2	0.8511	0.0144	0.1024	0.0260	0.2836	0.2012	<0.9473
Least-quantifiable-20-uniform-3	0.8889	0.0099	0.0957	0.0263	0.2734	0.2166	<0.9473
Least-20-genera-uniform-1	1.0	0.0180	0.9474	0.9474	0.9744	0.5000	<0.6154
Least-20-genera-uniform-2	0.9091	0.0105	0.0978	0.0193	0.2667	0.2500	<0.6000
Least-20-genera-uniform-3	0.9302	0.0144	0.0974	0.0192	0.2535	0.2344	<0.6316
Least-20-genera-lognormal	0.7547	0.0081	0.0902	0.0185	0.3091	0.1871	<0.6000
Random-20-uniform	1.0	0.2628	0.9756	0.9524	0.8696	0.2093	<0.5500
Random-20-lognormal	0.9756	0.6061	0.9524	0.9302	0.8333	0.5797	<0.4865
Random-20-lognormal-a.g.	1.0	0.6909	0.9091	0.9091	0.9302	0.7170	<0.7647
Random-100-uniform	1.0	0.9851	0.9950	0.9950	0.9852	0.9662	<0.8085
HumanGut-least-25	0.9412	0.2396	0.7692	0.7692	0.6757		∼0.8182
HumanGut-random-100-1	0.9950	0.5513	0.9851	0.9950	0.9340	N/A	∼0.8087
HumanGut-random-100-2	0.9901	0.6263	0.9798	0.9899	0.8959		∼0.7791
HumanGut-all	0.9902	0.9500	0.9801	0.9839	0.9739		∼0.7815

Supplementary Table 5: The F1 score of CAMMiQ, Kraken2, KrakenUniq, CLARK, Centrifuge, Bracken and MetaPhlAn2 on all `species-level-all`, `species-level-bacteria` and `strain-level` queries, for genome identification.

6.9 Genome Identification Threshold for Kraken2, KrakenUniq, Centrifuge, CLARK and Bracken

To minimize the number of false positives and allow a fair comparison to CAMMiQ, we filtered out genomes reported by Kraken2, KrakenUniq, Centrifuge, and Bracken with read count less than 0.0001 times the total number of reads assigned to species level, and reported the number of remaining genomes in **Table 3** and **Table 5**, main text. For CLARK, we filtered out genomes with read count less than 0.0001 times the total number of read assignments. In addition, since genomes in our `strain-level` index dataset sometimes have species level taxonomic IDs (which will lead to a species-level assignment for these tools), we used the total number of reads assigned to **any taxonomic ID** in the `strain-level` index dataset as the total strain level read counts to do the filtering. This process resulted in significantly less false positives. However, it may also lead to a miss of true positive genomes, as per `CAMMiQ`.

7 Alternative Performance Metrics for Genome Identification and Quantification

7.1 F1 Score for Genome Identification

The F1 score for genome identification in **Supplementary Table 5** was computed as

$$\text{F1} = \frac{2 \cdot \text{Genome Identification Precision} \cdot \text{Genome Identification Recall}}{\text{Genome Identification Precision} + \text{Genome Identification Recall}}.$$

Note that for MetaPhlAn2 at species level, as we actually used the genus level identifications to compute the precision and recall (there should be less true positive counts at specie level), we may overestimate its F1 score. As such we used its genus level F1 score as an upper bound of its species level score, and marked '$<$' in front of the values. However, at strain level, it is possible for both missing true positive identifications, and 'false' true positive identifications due to matching species/strain names. Therefore, the resulting F1 score might be inaccurate, and are marked with '$\sim$' in front of the values.

8 `CAMMiQ` Parameters

As a part of our performance benchmark, we finally evaluated different choices on two major parameters that can impact `CAMMiQ`'s accuracy performance on read classification and genome identification and quantification: α, the minimum relative read count threshold for reporting a genome, and $L_{\min}$, the minimum unique or double-unique substring length (values larger than the default value of 50 for $L_{\max}$ did not have a big impact and thus are not reported here). Note that in `CAMMiQ` implementations we also allow users to adjust other parameters, e.g. h, the length of the prefix of substrings that is not chained in the hash table; and $L_{\max}$, the maximum length of a unique or doubly unique substring that is hashed. Since the value of h does not impact which substrings are hashed but rather alters how they are hashed, it only impacts speed, but not accuracy. In contrast, as $L_{\max}$ increases, the number of unique substrings in a genome and thus the accuracy of `CAMMiQ` should improve. In practice, however, we observe very few unique and doubly unique substrings between $L/2$ (`CAMMiQ`'s default setting for $L_{\max}$) and L, where L is the read length in a query. Therefore, `CAMMiQ`'s performance was minimally impacted after increasing the value of $L_{\max}$, for any of the four index data sets we used. Since these are good representatives of an index data set to be used in practice, we believe setting $L_{\max}$ to half of the read length is a good rule of thumb.

In **Supplementary Table 6** we report the results for each possible combination of $L_{\min} = 21, 26, 31$ and $\alpha = 0.001, 0.0001$, on 8 of the 11 queries, omitting the 3 error free queries (on which the impact on classification precision is minimal). As we increase $L_{\min}$, `CAMMiQ`'s classification precision improves, however its read assignment performance (classification recall) deteriorates. Interestingly, its predicted abundance values did not change much with increasing $L_{\min}$. As a result we set the default $L_{\min}$ to 26 in `CAMMiQ`. On the other hand, increasing the value of α, decreased the number of false positives in `CAMMiQ`'s output, particularly in the most difficult queries. However, as a result of this, for the queries Least-20-genera-lognormal and Random-20-lognormal-a.g., those genomes with low abundance values were disregarded by `CAMMiQ`, leading to false negatives. `CAMMiQ` allows the user to set the parameter α with prior knowledge on the reads to be queried (e.g., the expected read coverage or the number of genomes in the query); we set its default value to 0.0001.

9 blastn analysis of *Salmonella* strains

The RNASeq reads for each cell studied in [15] are stored as separate read sets in the Sequence Read Archive (SRA) [24]. To reduce the number of reads that need to be downloaded or aligned, we used ReadFinder (https://github.com/morgulis/ReadFinder) to find any reads that could plausibly map to either the *Salmonella* strains *LT2* or *D23580* with permissive parameters allowing alignments that stray by as much as four diagonals from the main diagonal. ReadFinder uses similar methods as SRPRISM [25] to search SRA without the need to download the data and is more streamlined than SRPRISM for our purpose of finding candidate matching reads. We then used blastn [26] with word size 16 to find local (and ideally,

Query Set	$L_{\min}$	Classif-ication Precision	Num. Assi-gned Reads	Num. Identified Species		L1 Err.	
				$\alpha = 0.001$	$\alpha = 0.0001$	$\alpha = 0.001$	$\alpha = 0.0001$
Least-20-uniform	21	0.887	1.69M	20/21	31/32	0.0835	0.0836
	26	0.974	1.57M	20/21	28/29	0.0929	0.0929
	31	0.980	1.49M	20/20	22/23	0.1064	0.1060
Least-quantifiable-20-uniform	21	0.967	2.85M	20/20	29/31	0.0423	0.0400
	26	0.989	2.81M	20/20	27/29	0.0294	0.0278
	31	0.992	2.70M	20/20	24/26	0.0344	0.0326
Least-20-genera-uniform	21	0.974	2.61M	20/20	25/26	0.0706	0.0715
	26	0.993	2.58M	20/20	24/26	0.0585	0.0591
	31	0.995	2.48M	20/20	23/24	0.0688	0.0683
Least-20 genera-lognormal	21	0.974	2.56M	19/19	34/35	0.0394	0.0418
	26	0.993	2.53M	19/19	33/34	0.0416	0.0439
	31	0.995	2.43M	19/19	32/33	0.0355	0.0374
Random-20-uniform	21	0.993	3.83M	20/20	20/20	0.0220	0.0220
	26	0.997	3.84M	20/20	20/20	0.0113	0.0113
	31	0.998	3.69M	20/20	20/20	0.0294	0.0294
Random-20-lognormal	21	0.996	4.50M	20/20	21/21	0.0432	0.0431
	26	0.998	4.48M	20/20	21/21	0.0039	0.0038
	31	0.998	4.32M	20/20	21/21	0.0062	0.0058
Random-20-lognormal-a.g.*	21	0.989	0.92M	17/17	20/20	0.1492	0.1268
	26	0.998	0.91M	16/16	20/20	0.1631	0.1262
	31	0.999	0.87M	16/16	20/20	0.1658	0.1298
Random-100-uniform	21	0.996	19.5M	100/100	100/100	0.0176	0.0176
	26	0.998	19.5M	100/100	100/100	0.0096	0.0096
	31	0.999	19.0M	100/100	100/100	0.0104	0.0104

Supplementary Table 6: Performance of `CAMMiQ` as a function of minimum unique/doubly-unique substring length $L_{\min} = 21, 26, 31$, and minimum relative read count threshold $\alpha = 0.001, 0.0001$ to report a genome. Classification Precision: the proportion of reads correctly assigned to a genome among the set of reads assigned to some genome, correctly or incorrectly. Number of assigned reads: the total number of reads assigned to some genome. Number of identified genomes: the number of genomes returned by `CAMMiQ`'s ILP formulation v.s. the number of genomes that have sufficient read assignments (determined by α). L1 error: the L1 distance between the true relative abundance values (between 0 and 1) and the predicted abundance values for each genome in the corresponding query.
*: 10% reads in the query Random-20-lognormal-a.g. are from a genome not in the index; any assignment of such a read to a genome is necessarily incorrect.

global) alignments between the reads identified by ReadFinder and either *Salmonella* strain. This test is much simpler than the `CAMMiQ` test because we did not consider alignments to any *Salmonella* strains other than the two actually used in the original wet lab experiment.

Most reads that align to either strain actually align to the two strains equally well. To decide which reads align strictly better to one strain or the other, we implemented an in-house script with the following rules. A blastn alignment is "high-quality " if it has length at least 70 (taking into account that the reads are of length 75), has identity percentage ≥ 98. A read R maps better to the *LT2* strain if either:

- R has a high-quality alignment to the *LT2* strain but R has no high-quality alignment to the *D23580* strain or
- R has high-quality alignments to both strains and the best *LT2* alignment is at least as good as the best *D23580* alignment on i) length ii) identity percentage, iii) gaps and is better than the best D-strain alignment on at least one of the thee Roman numeral criteria.

The criteria for mapping better to the D-strain are symmetric. The script reports counts of how many reads map align strictly better to each of the two strains. Although the data in [15] consist of paired reads, each mate pair was treated individually in the blastn analysis.

References

[1] Matias, Y., Muthukrishnan, S., Sahinalp, S. C. & Ziv, J. Augmenting suffix trees, with applications. In *European Symposium on Algorithms*, 67–78 (Springer, 1998).

[2] Kasai, T., Lee, G., Arimura, H., Arikawa, S. & Park, K. Linear-time longest-common-prefix computation in suffix arrays and its applications. In *Annual Symposium on Combinatorial Pattern Matching*, 181–192 (Springer, 2001).

[3] Kärkkäinen, J. & Sanders, P. Simple linear work suffix array construction. In *International Colloquium on Automata, Languages, and Programming*, 943–955 (Springer, 2003).

[4] Ilie, L. & Smyth, W. F. Minimum unique substrings and maximum repeats. *Fundamenta Informaticae* **110**, 183–195 (2011).

[5] Weissman, T., Ordentlich, E., Seroussi, G., Verdu, S. & Weinberger, M. J. Inequalities for the L1 deviation of the empirical distribution. *Hewlett-Packard Labs, Tech. Rep* (2003).

[6] Pruitt, K. D., Tatusova, T. & Maglott, D. R. NCBI reference sequences (RefSeq): a curated non-redundant sequence database of genomes, transcripts and proteins. *Nucleic Acids Research* **35**, D61–D65 (2007).

[7] Simon, H. Y., Siddle, K. J., Park, D. J. & Sabeti, P. C. Benchmarking metagenomics tools for taxonomic classificationspa. *Cell* **178**, 779–794 (2019).

[8] Wood, D. E., Lu, J. & Langmead, B. Improved metagenomic analysis with Kraken 2. *Genome Biology* **20**, 257 (2019).

[9] Wood, D. E. & Salzberg, S. L. Kraken: ultrafast metagenomic sequence classification using exact alignments. *Genome Biology* **15**, R46 (2014).

[10] Breitwieser, F., Baker, D. & Salzberg, S. L. KrakenUniq: confident and fast metagenomics classification using unique k-mer counts. *Genome Biology* **19**, 198 (2018).

[11] Ounit, R., Wanamaker, S., Close, T. J. & Lonardi, S. Clark: fast and accurate classification of metagenomic and genomic sequences using discriminative k-mers. *BMC Genomics* **16**, 236 (2015).

[12] Kim, D., Song, L., Breitwieser, F. P. & Salzberg, S. L. Centrifuge: rapid and sensitive classification of metagenomic sequences. *Genome Research* **26**, 1721–1729 (2016).

[13] Lu, J., Breitwieser, F. P., Thielen, P. & Salzberg, S. L. Bracken: estimating species abundance in metagenomics data. *PeerJ Computer Science* **3**, e104 (2017).

[14] Forster, S. C. *et al.* A human gut bacterial genome and culture collection for improved metagenomic analyses. *Nature Biotechnology* **37**, 186 (2019).

[15] Aulicino, A. *et al.* Invasive *Salmonella* exploits divergent immune evasion strategies in infected and bystander dendritic cell subsets. *Nature Communications* **9**, 4883 (2018).

[16] Walker, M. A. *et al.* GATK PathSeq: a customizable computational tool for the discovery and identification of microbial sequences in libraries from eukaryotic hosts. *Bioinformatics* **34**, 4287–4289 (2018).

[17] McIntyre, A. B. R. *et al.* Comprehensive benchmarking and ensemble approaches for metagenomic classifiers. *Genome Biology* **18**, 72 (2017).

[18] Huang, W., Li, L., Myers, J. R. & Marth, G. T. Art: a next-generation sequencing read simulator. *Bioinformatics* **28**, 593–594 (2012).

[19] Afshinnekoo, E. *et al.* Geospatial resolution of human and bacterial diversity with city-scale metagenomics. *Cell Systems* **1**, 72–87 (2015).

[20] Ounit, R. & Lonardi, S. Higher classification sensitivity of short metagenomic reads with clark-s. *Bioinformatics* **32**, 3823–3825 (2016).

[21] Gourlé, H., Karlsson-Lindsjö, O., Hayer, J. & Bongcam-Rudloff, E. Simulating illumina metagenomic data with insilicoseq. *Bioinformatics* **35**, 521–522 (2019).

[22] Dobin, A. *et al.* Star: ultrafast universal rna-seq aligner. *Bioinformatics* **29**, 15–21 (2013).

[23] Robinson, W., Schischlik, F., Gertz, E. M., Schäffer, A. A. & Ruppin, E. Identifying the landscape of intratumoral microbes via a single cell transcriptomic analysis. *bioRxiv* (2020).

[24] Leinonen, R., Sugawara, H., Shumway, M. *et al.* The sequence read archive. *Nucleic Acids Research* **39**, D19–D21 (2011).

[25] Morgulis, A. & Agarwala, R. SRPRISM (Single Read Paired Read Indel Substitution Minimizer): an efficient aligner for assemblies with explicit guarantees. *GigaScience* **9**, giaa023 (2020).

[26] Altschul, S. F. *et al.* Gapped BLAST and PSI-BLAST: a new generation of protein database search programs. *Nucleic Acids Research* **25**, 3389–3402 (1997).
