# Supplementary material for: Strain level microbial detection and quantification with applications to single cell metagenomics
Source: Nat Commun. 2022 Oct 28;13:6430. doi: 10.1038/s41467-022-33869-7 (PMC9616933; doi:10.1038/s41467-022-33869-7)
Supplement: Supplementary file 3 — Reporting Summary [file 41467_2022_33869_MOESM3_ESM.pdf]

nature portfolio

Corresponding author(s): S. Cenk Sahinalp

Last updated by author(s): 2022/09/27

Reporting Summary

Nature Portfolio wishes to improve the reproducibility of the work that we publish. This form provides structure for consistency and transparency in reporting. For further information on Nature Portfolio policies, see our Editorial Policies and the Editorial Policy Checklist.

Statistics

For all statistical analyses, confirm that the following items are present in the figure legend, table legend, main text, or Methods section.

n/a	Confirmed	
☐	☒	The exact sample size (*n*) for each experimental group/condition, given as a discrete number and unit of measurement
☒	☐	A statement on whether measurements were taken from distinct samples or whether the same sample was measured repeatedly
☐	☒	The statistical test(s) used AND whether they are one- or two-sided<br>*Only common tests should be described solely by name; describe more complex techniques in the Methods section.*
☒	☐	A description of all covariates tested
☒	☐	A description of any assumptions or corrections, such as tests of normality and adjustment for multiple comparisons
☐	☒	A full description of the statistical parameters including central tendency (e.g. means) or other basic estimates (e.g. regression coefficient) AND variation (e.g. standard deviation) or associated estimates of uncertainty (e.g. confidence intervals)
☐	☒	For null hypothesis testing, the test statistic (e.g. *F*, *t*, *r*) with confidence intervals, effect sizes, degrees of freedom and *P* value noted<br>*Give P values as exact values whenever suitable.*
☒	☐	For Bayesian analysis, information on the choice of priors and Markov chain Monte Carlo settings
☒	☐	For hierarchical and complex designs, identification of the appropriate level for tests and full reporting of outcomes
☒	☐	Estimates of effect sizes (e.g. Cohen's *d*, Pearson's *r*), indicating how they were calculated

Our web collection on statistics for biologists contains articles on many of the points above.

Software and code

Policy information about availability of computer code

Data collection	All (complete) bacterial, viral and archaeal genomes from RefSeq used in this paper can be downloaded with python script CAMMiQ-download; the four (species-level-all, species-level-bacteria, strain-level and subspecies-level) index datasets correspond to different subsets of RefSeq genomes; queries simulated on RefSeq or a subset of RefSeq genomes were obtained by running the python script CAMMiQ-simulate; both scripts are provided as a part of CAMMiQ software https://github.com/algo-cancer/CAMMiQ.<br><br>No preprocessing steps were performed on the CAMI and IMMSA queries.<br><br>To obtain our scRNA-seq queries, a quality control filtering step was first done by Aulicino et al., associated with the data in NCBI Bioproject PRJNA437328. This step filtered out reads from 31 cells - Dr. Aulicino and Dr. Robinson had direct communication in August 2020 regarding which cells Dr. Aulicino recommended to filter out. Then, the reads from the remaining 342 cells were aligned to the reference human genome using STAR aligner. Finally, we remove all mapped reads and use the remaining as our scRNA-seq queries.
Data analysis	The scripts for generating our index datasets and queries are available at https://github.com/algo-cancer/CAMMiQ. In addition, for compiling out scRNA-seq queries, STAR aligner is available at https://github.com/alexdobin/STAR.<br><br>We compare the performance of CAMMiQ against Kraken2, KrakenUniq, Centrifuge, Bracken, CLARK, MetaPhlAn2 and GATK Pathseq. Kraken2 software is publicly available at https://github.com/DerrickWood/kraken2; KrakenUniq software is publicly available at https://github.com/fbreitwieser/krakenuniq; Centrifuge software is publicly available at https://github.com/DaehwanKimLab/centrifuge; Bracken is publicly available at https://github.com/jenniferlu717/Bracken; CLARK is publicly available at http://clark.cs.ucr.edu; MetaPhlAn2 is publicly available at https://github.com/biobakery/MetaPhlAn2 (note that MetaPhlAn2 requires bowtie2, which is publicly available at https://github.com/BenLangmead/bowtie2; currently the list of marker genes can be found at https://figshare.com/articles/dataset/db_v20/6200807); GATK Pathseq is publicly available at https://github.com/gatk-workflows/gatk4-pathseq.

Finally, on scRNA-seq queries, we also compare CAMMiQ with blastn, for that we first used ReadFinder (https://github.com/morgulis/ReadFinder) to find any reads that could plausibly map to either the Salmonella strains LT2 or D23580 and then used blastn (https://blast.ncbi.nlm.nih.gov) with word size 16 to find local alignments between the reads identified by ReadFinder and either Salmonella strain.

For manuscripts utilizing custom algorithms or software that are central to the research but not yet described in published literature, software must be made available to editors and reviewers. We strongly encourage code deposition in a community repository (e.g. GitHub). See the Nature Portfolio guidelines for submitting code & software for further information.

Data

Policy information about availability of data

All manuscripts must include a data availability statement. This statement should provide the following information, where applicable:

- Accession codes, unique identifiers, or web links for publicly available datasets
- A description of any restrictions on data availability
- For clinical datasets or third party data, please ensure that the statement adheres to our policy

There are four index datasets (species-level-all, species-level-bacteria, strain-level and subspecies-level) and associated query sets used in this paper. All of the four index datasets include a subset of all (complete) bacterial, viral and archaeal genomes from NCBI's RefSeq database, which is available at is available at ftp://ftp.ncbi.nlm.nih.gov/genomes/refseq.

For the species-level-all index dataset, we use the release version 205 of RefSeq, which can be found at https://ftp.ncbi.nlm.nih.gov/refseq/release/release-notes/archive/RefSeq-release205.txt. The complete list of 16418 genomes can be found in https://github.com/algo-cancer/CAMMiQ/blob/master/README.md. The corresponding IMMSA queries can be found privately at http://ftp-private.ncbi.nlm.nih.gov/nist-immsa/IMMSA. A publicly available copy of the above directory is available at https://ftp.ncbi.nlm.nih.gov/pub/catSMA/for_Kaiyuan. The CAMI queries as well as the ground truth files can be found at at http://gigadb.org/dataset/100344.

For the species-level-bacteria index dataset, we use the release version 93 of RefSeq, which can be found at https://ftp.ncbi.nlm.nih.gov/refseq/release/releasenotes/archive/RefSeq-release93.txt. The complete list of 4122 genomes can be found in https://github.com/algo-cancer/CAMMiQ/blob/master/README.md. The corresponding queries were generated by a python script CAMMiQ-simulate, which is available along with the software repo https://github.com/algo-cancer/CAMMiQ; these queries are available upon request.

For the strain-level index dataset, we use the release version 93 of RefSeq, which can be found at https://ftp.ncbi.nlm.nih.gov/refseq/release/release-notes/archive/RefSeq-release93.txt. The list of human gut associated bacteria was obtained in the Supplementary Table 1 from https://www.nature.com/articles/s41587-018-0009-7. The complete list of 614 genomes can be found in https://github.com/algo-cancer/CAMMiQ/blob/master/README.md. The corresponding queries were also generated by running CAMMiQ-simulate, which is available along with the software repo https://github.com/algo-cancer/CAMMiQ; these queries are available upon request.

For our subspecies-level index dataset, we use the release version 93 of RefSeq, which can be found at https://ftp.ncbi.nlm.nih.gov/refseq/release/release-notes/archive/RefSeq-release93.txt. The complete list of 3395 genomes can be found in https://github.com/algo-cancer/CAMMiQ/blob/master/README.md. The corresponding scRNA-seq queries can be obtained from https://www.ncbi.nlm.nih.gov/bioproject/PRJNA437328.

Field-specific reporting

Please select the one below that is the best fit for your research. If you are not sure, read the appropriate sections before making your selection.

[x] Life sciences [ ] Behavioural & social sciences [ ] Ecological, evolutionary & environmental sciences

For a reference copy of the document with all sections, see nature.com/documents/nr-reporting-summary-flat.pdf

Life sciences study design

All studies must disclose on these points even when the disclosure is negative.

Sample size	The species-level-all index dataset includes 16418 genomes. The species-level-bacteria index dataset includes 4122 genomes. The strain-level index dataset includes 614 genomes. The subspecies-level index dataset includes 3395 genomes. The size of these four index datasets were determined by specific version(s) of RefSeq. Each of the 8 CAMI queries contains roughly 99.8 million 150bp reads. The number of reads in the 8 IMMSA queries varies from 0.5 million to 5.7 million, with read length fixed to 100bp. There queries were obtained from earlier benchmarks. Each of the CAMMiQ simulated queries at species or strain level contain 1.1 million to 21.5 million 100-125bp reads, matching the size of a typical metagenomic dataset. The 342 single cell RNA-seq queries were obtained from an earlier study, giving in total 8.5 million reads with length 66.4bp on average and standard deviation 13.6.
Data exclusions	In our strain-level index dataset, we excluded 3 genomes in Supplementary Table 1 from https://www.nature.com/articles/s41587-018-0009-7 because they were not included in release 93 of the RefSeq database.<br><br>In our scRNA-seq queries, we excluded the reads in 31 cells because these cells did not pass the quality filtering step described in "Data collection" section of this form.
Replication	Our computational experiments can be reliably reproduced by installing and running the CAMMiQ software on typical Linux servers.
Randomization	N/A

Blinding	N/A

Reporting for specific materials, systems and methods

We require information from authors about some types of materials, experimental systems and methods used in many studies. Here, indicate whether each material, system or method listed is relevant to your study. If you are not sure if a list item applies to your research, read the appropriate section before selecting a response.

Materials & experimental systems

n/a	Involved in the study
☒	☐ Antibodies
☒	☐ Eukaryotic cell lines
☒	☐ Palaeontology and archaeology
☒	☐ Animals and other organisms
☒	☐ Human research participants
☒	☐ Clinical data
☒	☐ Dual use research of concern

Methods

n/a	Involved in the study
☒	☐ ChIP-seq
☒	☐ Flow cytometry
☒	☐ MRI-based neuroimaging
